# Maternal cigarette smoking before and during pregnancy and the risk of preterm birth: A dose–response analysis of 25 million mother–infant pairs

**DOI:** 10.1371/journal.pmed.1003158

**Published:** 2020-08-18

**Authors:** Buyun Liu, Guifeng Xu, Yangbo Sun, Xiu Qiu, Kelli K. Ryckman, Yongfu Yu, Linda G. Snetselaar, Wei Bao

**Affiliations:** 1 Department of Epidemiology, College of Public Health, University of Iowa, Iowa City, Iowa, United States of America; 2 Division of Birth Cohort Study, Guangzhou Women and Children’s Medical Center, Guangzhou Medical University, Guangzhou, China; 3 Department of Epidemiology, Fielding School of Public Health, University of California, Los Angeles, California, United States of America; 4 Department of Clinical Epidemiology, Aarhus University, Aarhus, Denmark; University of Edinburgh, UNITED KINGDOM

## Abstract

**Background:**

Most of the women who smoke before pregnancy continue smoking during pregnancy, and some start to quit smoking after being pregnant, although existing guidelines for pregnancy recommend that women who smoke should quit smoking before pregnancy. Findings about the timing and intensity of maternal smoking, especially low-intensity smoking (1–9 cigarettes per day), and preterm birth are still inconsistent and ambiguous. This study aimed to examine the association of the timing of smoking and doses of smoking before pregnancy and during the first or second trimester of pregnancy with preterm birth in a large-scale population-based retrospective cohort study.

**Methods and findings:**

We used nationwide birth certificate data from singleton mother–infant pairs in the United States National Vital Statistics System, 2011–2018. All adult women with live singleton births, without preexisting hypertension or diabetes, and with complete data on smoking and gestational age at delivery were included. Participants reported their smoking status (yes or no) and daily number of cigarettes consumed before and during each trimester of pregnancy. The outcome of interest was preterm birth, defined as a birth before 37 weeks of gestation. Logistic regression models were used to estimate the odds ratio (OR) with 95% confidence intervals (CIs) of preterm birth associated with smoking status and the number of cigarettes consumed, adjusting for maternal age, race/ethnicity, parity, education levels, prepregnancy BMI, previous history of preterm birth, marital status, infant sex, and initiation of prenatal care. This study included 25,623,479 women, with a mean age of 29 years (range 20–50 years); 13,742,486 (53.6%) participants were of non-Hispanic white ancestry, 5,971,598 (23.3%) of Hispanic ancestry, and 3,417,456 (13.34%) of non-Hispanic black ancestry. The prevalence of preterm birth was 9.3% (*n* = 2,378,398). We found that maternal smoking during pregnancy, even at a very low level of intensity, was associated with an increased risk of preterm delivery. The adjusted ORs (95% CI) of preterm birth for mothers who smoked 1–2, 3–5, 6–9, 10–19, and ≥20 cigarettes per day during the first trimester compared with mothers who did not smoke were 1.31 (1.29–1.33), 1.31 (1.30–1.32), 1.33 (1.31–1.35), 1.44 (1.43–1.45), and 1.53 (1.52–1.55), respectively (all *P* values < 0.001), whereas for those who smoked during the second trimester, the corresponding ORs were 1.37 (1.35–1.39), 1.36 (1.35–1.38), 1.36 (1.34–1.38), 1.48 (1.47–1.49), and 1.59 (1.58–1.61), respectively (all *P* values < 0.001). Furthermore, smokers who quit before pregnancy, regardless of smoking intensity, had a comparable risk of preterm birth with nonsmokers, although this was not the case when cessation occurred in the first or second trimester of pregnancy. The major limitation of this study is the self-reported information about smoking, which may be subject to information bias. In addition, we cannot rule out the possibility of residual confounding caused by unmeasured factors in an observational research design.

**Conclusions:**

In this study, we observed that low-intensity cigarette consumption during either the first or second trimester of pregnancy, even as low as 1–2 cigarettes per day, was associated with an increased risk of preterm birth. These findings suggest that there is no safe level or safe trimester for maternal smoking during pregnancy. Women of reproductive age who smoke should be strongly encouraged and supported to quit smoking before pregnancy.

## Abstract 2

Buyun Liu and colleagues study maternal smoking and risk of preterm birth in the United States

## Author summary

**Why was this study done?:**

- It is well known that smoking during pregnancy is associated with increased risk of adverse birth outcomes, including preterm birth.
- However, the associations between the timing and the intensity of maternal smoking, especially low-intensity cigarette smoking (e.g., 1–9 cigarettes per day), and preterm birth are unclear.

**What did the researchers do and find?:**

- In a large-scale population-based retrospective cohort study consisting of 25,623,479 mothers, we examined the association of the timing of smoking and doses of smoking before pregnancy and during the first or second trimester of pregnancy with preterm birth.
- Maternal cigarette smoking during either the first or the second trimester of pregnancy, even as low as 1–2 cigarettes per day, was associated with increased risks of preterm birth.
- Smokers who quit before pregnancy, regardless of smoking intensity, had a comparable risk of preterm birth with nonsmokers, although this was not the case when cessation occurred in the first or second trimester of pregnancy.

**What do these findings mean?:**

- Our findings suggest that no safe level or safe trimester exists for maternal smoking during pregnancy.
- Public health efforts should be made to help women who smoke stop smoking completely before pregnancy to prevent preterm delivery.

## Introduction

Preterm birth is not only the leading cause of mortality in children under 5 years old worldwide [1] but also associated with long-term adverse health outcomes in children’s later life [2]. The most recent report showed that the estimated global rate of preterm birth increased from 9.8% (12,658,588) in 2000 to 10.6% (14,835,606) in 2014 [3]. In the US, the rate of preterm birth rose from 9.6% (381,321 in 3,988,076 births) in 2014 to 10.02% (379,777 in 3,791,712 births) in 2018 after a continuous decline from 2006 to 2013 [4]. A previous estimation by the US Institute of Medicine showed that the annual societal economic cost associated with preterm birth in the US was US$26.2 billion in 2005 [5]. Therefore, identifying the potential causes and effective prevention of preterm birth remains a public health priority in the United States and worldwide [6].

Maternal cigarette smoking during pregnancy is known as one of the most important modifiable causes of poor maternal and fetal outcomes [7]. Although efforts have been made to control smoking during pregnancy [8], it is still prevalent in many countries [9]. In the US, one in 14 women smoked during pregnancy in 2016 [10]. This may, partially, be attributed to the belief that light smoking carries little or no harm [11]. Although existing guidelines for pregnancy recommend women who smoke to quit smoking before pregnancy, most of those women who smoke before pregnancy continue to smoke during pregnancy or start to quit smoking after becoming pregnant [12]. Findings about the timing and the intensity of maternal smoking and preterm birth are still inconsistent and ambiguous. Some recent studies even showed that smoking during a specific period or at low levels might not be as harmful as expected, which provides conflicting advice. For example, previous studies found that maternal smoking during only early pregnancy (first or second trimester) did not increase the risk of preterm delivery [13–15]. Other studies reported that women who quit smoking before the last 3 months of pregnancy had a similar risk of preterm birth compared with those who never smoke [16,17]. In terms of the intensity of smoking, although prenatal exposure to heavy cigarette consumption has been associated with preterm birth [15,16,18], little is known about the effects of low-level cigarette smoking, especially by specific trimester. A systematic review of six studies conducted before 2000 suggested that smoking 1–10 cigarettes per day during pregnancy was associated with increased risk of preterm birth [19]. However, subsequent research found that light smoking (1–9 cigarettes per day) during any of the three trimesters was not associated with preterm birth [12,20,21]. Other studies reported that light smoking during late pregnancy (the second or third trimester) was significantly associated with shorter gestational age [16,18]. Significant associations were also found for the first trimester [15]. To date, studies that fully include the effects of the timing and intensity of maternal smoking on preterm birth are notably lacking.

Given the high prevalence and life-threating impact of preterm birth and inconsistent findings from previous studies, a thorough and quantitative understanding of the potential effects of maternal smoking before and during pregnancy is urgently needed. Therefore, this study examined the association between maternal cigarette use before pregnancy and during the first and second trimesters of pregnancy and the risk of preterm birth by taking advantage of a nationwide large cohort in the US.

## Methods

We used nationwide birth certificate data from the US National Vital Statistics System (NVSS). In the US, state laws require birth certificates to be completed for all births, and federal law mandates national collection and publication of births and other vital statistics data. The NVSS, as the federal compilation of this data, is the result of the cooperation between the National Center for Health Statistics (NCHS) at the Centers for Disease Control and Prevention (CDC) and all US states to provide access to statistical information from birth certificates. Standard forms for the collection of the data and model procedures for the uniform registration of the events are developed and recommended for state use through cooperative activities of the states and NCHS. To collect data from the best sources, two worksheets, including the mother’s worksheet and the facility worksheet, were developed for the 2003 revision of the US Standard Certificate of Live Birth. In the mother’s worksheet, data (e.g., race, education, etc.) are directly obtained from the mother; in the facility worksheet, data (e.g., such as prenatal care, prenatal complications, etc.) are obtained directly from the medical records. The NVSS data have been widely used in annual vital statistics reports by the CDC [22], other reports by the CDC [10], and the literature [23]. A detailed description of NVSS data collection methodology, quality control, vital statistics, and data access is available on the official website (https://www.cdc.gov/nchs/nvss/births.htm).

**Fig 1:**
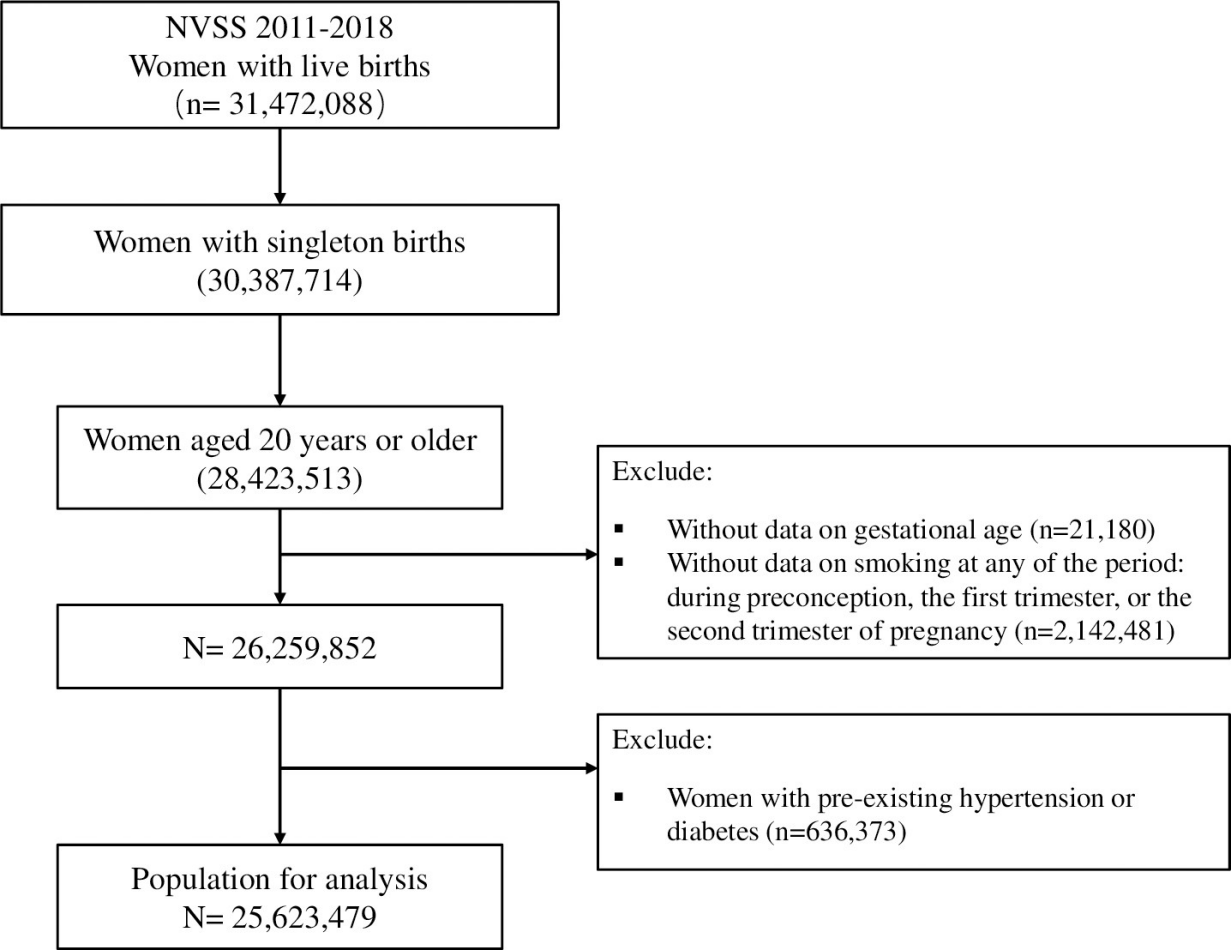
Participant flow chart. NVSS, National Vital Statistics System.

In the present study, we used NVSS data from 2011–2018 because some important information, such as maternal prepregnancy BMI, was collected since 2011. We included all live singleton births (*n* = 26,259,852) documented in NVSS from 2011 to 2018 if the mothers were aged 20 years or older and have complete data on smoking before and during pregnancy and gestational age. Mothers with preexisting hypertension or diabetes were excluded (*n* = 636,373) because these diseases are strong risk factors for adverse birth outcomes, including preterm birth. The final sample consisted of 25,623,479 live singleton births. A participant flow chart was provided (**Fig 1**).

This study followed the reporting guidelines in the Strengthening the Reporting of Observational Studies in Epidemiology (STROBE) for cohort study (S1 STROBE Checklist). Because these data are deidentified, this study was exempt from institutional review board approval. The analyses were performed from November 2018 to May 2019. There was no documented protocol for the analysis.

### Exposure measurement

In this study, our exposure variables of interest were maternal cigarette smoking 3 months before pregnancy and during early pregnancy (first or second trimester). We did not use data on maternal cigarette smoking during the third trimester of pregnancy, because most preterm deliveries occur during the third trimester, which complicates the exposure duration of maternal smoking during this period [24].

The information about smoking was collected using the mother’s worksheet from the 2003 revised birth certificate in the hospital around the time of delivery [10]. Mothers reported the average daily number of cigarettes or packs they consumed in the 3 months before pregnancy and in each trimester. Mothers who smoked “0” cigarettes were classified as nonsmokers, whereas those who smoked one or more cigarettes per day in the 3 months before pregnancy or in any of the three trimesters of pregnancy were classified as smokers in the specific period. To capture the natural variability of the timing of smoking pattern before and during pregnancy, participants were divided into eight groups according to their smoking status (yes or no) before pregnancy and during the first and the second trimesters of pregnancy [25]. According to the number of cigarettes smoked daily, smokers were grouped into 1–2, 3–5, 6–9, 10–19, or ≥20 cigarettes per day. Mothers with unknown smoking status for any of these periods were excluded from this study, as shown in **Fig 1**.

### Outcome measurement

Gestational age was calculated using the date of the last menstrual period (LMP) and the date of delivery. LMP was mainly obtained from hospital records or the physician. The mother would be asked for this information if data were unavailable from these resources. Imputation is done by the NCHS if the date of LMP is missing but the month and year are valid [26]. Preterm birth, defined as delivery occurring at less than 37 weeks of gestation, was the main outcome of interest [27]. We further subdivided the outcome into three types of preterm birth: extremely (<28 weeks), very (28–31 weeks), and moderately (32–36 weeks) preterm birth [27].

### Covariates assessment

Information about maternal demographics, parity, education, marital status, prepregnancy weight and height, prenatal care, and infant sex was collected using the maternal worksheet. Data on the previous history of preterm birth were obtained directly from the medical records using the facility worksheet.

In the analyses, we categorized the covariates as follows: maternal age (20–24, 25–29, 30–34, 35–39, or ≥40 years), race/ethnicity (Hispanic, non-Hispanic black, non-Hispanic white, or other [i.e., non-Hispanic American Indian/Native Alaskan, non-Hispanic Asian, non-Hispanic Native Hawaiians and other Pacific Islanders, non-Hispanic other races, non-Hispanic more than one race, origin unknown or not stated]), parity (1, 2, 3, or ≥4), maternal education (lower than high school, high school, or higher than high school), previous history of preterm birth (yes, no, or nulliparous), marital status (yes versus no), infant sex (boys versus girls), prepregnancy BMI (<18.5, 18.5–24.9, 25–29.9, 30–34.9, or ≥40 kg/m^2^), and timing of initiation of prenatal care (first to third, fourth to sixth, greater than seventh month, or no prenatal care). A missing category for covariates was added when necessary.

### Statistical analysis

Descriptive statistics about the population characteristics according to maternal smoking before and during pregnancy were presented. We performed a series of logistic regression models to estimate the odds ratios (ORs) with 95% confidence intervals (CIs) of preterm birth according to maternal cigarette smoking status. First, we estimated the association between the timing of maternal cigarette smoking and preterm birth with the mothers who never smoked before and during pregnancy as the reference group. A variety of potential confounders, including maternal age, race/ethnicity, parity, education levels, previous history of preterm birth, marital status, prepregnancy BMI, infant sex, and timing of initiation of prenatal care, were adjusted in the models. Second, we estimated the ORs of preterm birth according to the numbers of cigarettes consumed (0, 1–2, 3–5, 6–9, 10–19, ≥20 cigarettes per day) before and during pregnancy. We were especially interested in the association of low-intensity cigarette smoking with preterm birth. In addition, we repeated all the analyses for a finer categorization of preterm birth (i.e., extremely preterm birth, <28 weeks; very preterm birth, 28–31 weeks; moderately preterm birth, 32–36 weeks).

Moreover, we examined the association of smoking cessation at various periods during pregnancy with the risk of preterm birth. Cessation before pregnancy was defined as having smoked before pregnancy, but no smoking during the first trimester and the second trimester; cessation during the first trimester was defined as having smoked before pregnancy and during the first trimester, but no smoking during the second trimester; smoking throughout the second trimester was defined as having smoked 3 months before pregnancy and during the first and second trimesters of pregnancy; never smoking was defined as no smoking before or during pregnancy.

To evaluate potential effect modification, we conducted interaction and stratified analyses according to age (20–34 or ≥ 35 years), race/ethnicity (Hispanic, non-Hispanic white, non-Hispanic black, or other), and educational levels (lower than high school, high school, or higher than high school), an indicator of socioeconomic status, to explore the potential disparities of maternal age, race/ethnicity, and socioeconomic group in the association between maternal cigarette consumption and preterm birth. *P* values for heterogeneity were derived from the multiplicative interaction term coefficients (exposure variable × effect modifier variable) added to the main effects multivariable model. A sensitivity analysis was conducted using data from NVSS 2014–2018, in which the obstetric estimate of gestation at delivery was used to identify gestation age [28]. To further address the possibility of residual confounding, we also performed a sensitivity analysis by additionally adjusting for a propensity score that reflected associations of maternal smoking status with the covariates in the multivariate-adjusted model [29].

All statistical analyses were performed with SAS software (version 9.4; SAS Institute). Two-sided *P <* 0.05 was considered statistically significant.

## Results

This study included 25,623,479 mothers with live singleton births, with a mean age of 29 years (range 20–50 years); 13,742,486 (53.6%) participants were of non-Hispanic white ancestry, 5,971,598 (23.3%) of Hispanic ancestry, and 3,417,456 (13.34%) of non-Hispanic black ancestry. The prevalence of preterm birth was 9.3% (*n* = 2,378,398). Among them, 9.9% of mothers smoked before pregnancy, 7.4% smoked during the first trimester, and 6.4% smoked during the second trimester. Mothers who smoked before or during pregnancy were more likely to be younger, non-Hispanic white, less educated, unmarried, obese, and with higher parity (**Table 1**). Of the births, 2,378,398 (9.3%) were preterm birth. The prevalence of preterm birth according to population characteristics is shown in **S1 Table**.

**Table 1:** Population characteristics according to maternal smoking before and during pregnancy.

Variables	No. of participants	Smoking before pregnancy		Smoking during the first trimester		Smoking during the second trimester	
		No	Yes	No	Yes	No	Yes
Overall	25,623,479	23,087,504	2,535,975	23,718,917	1,904,562	23,983,690	1,639,789
Age, years, *n* (%)							
20–24	5,981,701	5,055,213 (21.9)	926,488 (36.5)	5,286,146 (22.3)	695,555 (36.5)	5,391,916 (22.5)	589,785 (36.0)
25–29	7,958,816	7,094,533 (30.7)	864,283 (34.1)	7,305,290 (30.8)	653,526 (34.3)	7,392,421 (30.8)	566,395 (34.5)
30–34	7,383,822	6,870,064 (29.8)	513,758 (20.3)	7,003,208 (29.5)	380,614 (20.0)	7,052,815 (29.4)	331,007 (20.2)
35–39	3,511,546	3,316,746 (14.4)	194,800 (7.7)	3,365,288 (14.2)	146,258 (7.7)	3,384,124 (14.1)	127,422 (7.8)
≥40	787,594	750,948 (3.3)	36,646 (1.5)	758,985 (3.2)	28,609 (1.5)	762,414 (3.2)	25,180 (1.5)
Race/ethnicity, *n* (%)							
Hispanic	5,971,598	5,798,379 (25.1)	173,219 (6.8)	5,865,973 (24.7)	105,625 (5.6)	5,890,457 (24.6)	81,141 (5.0)
Non-Hispanic white	13,742,486	11,806,776 (51.1)	1,935,710 (76.3)	12,259,933 (51.7)	1,482,553 (77.8)	12,447,836 (51.9)	1,294,650 (79.0)
Non-Hispanic black	3,417,456	3,123,271 (13.5)	294,185 (11.6)	3,197,688 (13.5)	219,768 (11.5)	3,233,682 (13.5)	183,774 (11.2)
Other	2,491,939	2,359,078 (10.2)	132,861 (5.2)	2,395,323 (10.1)	96,616 (5.1)	2,411,715 (10.1)	80,224 (4.9)
Education levels, *n* (%)							
Lower than high school	3,218,469	2,718,063 (11.8)	500,406 (19.7)	2,783,127 (11.7)	435,342 (22.9)	2,818,578 (11.8)	399,891 (24.4)
High school	6,147,452	5,155,920 (22.3)	991,532 (39.1)	5,366,789 (22.6)	780,663 (41.0)	5,466,076 (22.8)	681,376 (41.6)
Higher than high school	15,960,302	15,213,521 (64.7)	1,030,311 (40.6)	15,283,091 (64.4)	677,211 (35.6)	15,411,990 (64.3)	548,312 (33.4)
Missing	297,256	283,530 (1.2)	13,726 (0.5)	285,910 (1.2)	11,346 (0.6)	287,046 (1.2)	10,210 (0.6)
Marital status, *n* (%)							
Married	10,196,846	9,696,442 (42.0)	500,404 (19.7)	9,853,312 (41.5)	343,534 (18.0)	9,904,763 (41.3)	292,083 (17.8)
Unmarried	6,022,000	4,946,355 (21.4)	1,075,645 (42.4)	5,178,601 (21.8)	843,399 (44.3)	5,293,074 (22.1)	728,926 (44.5)
Missing	9,404,633	8,444,707 (36.6)	959,926 (37.9)	8,687,004 (36.6)	717,629 (37.7)	8,785,853 (36.6)	618,780 (37.7)
Parity, *n* (%)							
1	9,292,065	8,468,303 (36.7)	823,762 (32.5)	8,760,637 (36.9)	531,428 (27.9)	8,874,182 (37.0)	417,883 (25.5)
2	8,451,200	7,676,211 (33.3)	774,989 (30.6)	7,865,095 (33.2)	586,105 (30.8)	7,943,130 (33.1)	508,070 (31.0)
3	4,524,658	4,018,251 (17.4)	506,407 (20.0)	4,111,699 (17.3)	412,959 (21.7)	4,155,054 (17.3)	369,604 (22.5)
≥4	3,258,029	2,835,872 (12.3)	422,157 (16.7)	2,890,678 (12.2)	367,351 (19.3)	2,919,833 (12.2)	338,196 (20.6)
Missing	97,527	88,867 (0.4)	8,660 (0.3)	90,808 (0.4)	6,719 (0.4)	91,491 (0.4)	6,036 (0.4)
Timing of initiation of prenatal care, *n* (%)							
First to third month	367,407	17,516,056 (75.9)	1,656,724 (65.3)	17,991,743 (75.9)	1,181,037 (62.0)	18,172,623 (75.8)	1,000,157 (61.0)
Fourth to sixth month	19,172,780	3,689,591 (16.0)	576,684 (22.7)	3,799,676 (16.0)	466,604 (24.5)	3,859,936 (16.1)	406,344 (24.8)
Seventh to final month	4,266,280	926,529 (4.0)	158,410 (6.3)	951,172 (4.0)	133,767 (7.0)	964,595 (4.0)	120,344 (7.3)
No prenatal care	1,084,939	295,316 (1.3)	72,091 (2.8)	301,408 (1.3)	65,999 (3.5)	305,086 (1.3)	62,321 (3.8)
Missing	732,073	660,012 (2.9)	72,061 (2.8)	674,918 (2.9)	57,155 (3.0)	681,450 (2.8)	50,623 (3.1)
Previous history of preterm birth, *n* (%)							
Yes	729,606	603,586 (2.6)	126,020 (5.0)	624,224 (2.6)	105,382 (5.5)	634,681 (2.7)	94,925 (5.8)
No	15,581,179	13,998,464 (60.6)	1,582,715 (62.4)	14,316,529 (60.4)	1,264,650 (66.4)	14,457,127 (60.3)	1,124,052 (68.6)
Nulliparous	9,281,261	8,458,879 (36.6)	822,382 (32.4)	8,750,862 (36.9)	530,399 (27.9)	8,864,252 (37.0)	417,009 (25.4)
Missing	31,433	26,575 (0.1)	4,858 (0.2)	27,302 (0.1)	4,131 (0.2)	27,630 (0.1)	3,803 (0.2)
Prepregnancy BMI, kg/m^2^, *n* (%)							
<18.5	858,637	724,066 (3.1)	134,571 (5.3)	748,631 (3.2)	110,006 (5.8)	760,842 (3.2)	97,795 (6.0)
18.5–24.9	11,282,192	10,258,005 (44.4)	1,024,187 (40.4)	10,509,785 (44.3)	772,407 (40.6)	10,614,751 (44.3)	667,441 (40.7)
25–29.9	6,500,909	5,891,014 (25.5)	609,895 (24.1)	6,052,849 (25.5)	448,060 (23.5)	6,117,877 (25.5)	383,032 (23.4)
30–34.9	3,476,089	3,101,668 (13.4)	374,421 (14.8)	3,199,602 (13.5)	276,487 (14.5)	3,240,497 (13.5)	235,592 (14.4)
35–39.9	1,640,155	1,444,737 (6.3)	195,418 (7.7)	1,495,389 (6.3)	144,766 (7.6)	1,516,961 (6.3)	123,194 (7.5)
≥40	1,100,926	962,507 (4.2)	138,419 (5.5)	998,451 (4.2)	102,475 (5.4)	1,014,120 (4.2)	86,806 (5.3)
Missing	764,571	705,507 (3.1)	59,064 (2.3)	714,210 (3.0)	50,361 (2.6)	718,642 (3.0)	45,929 (2.8)
Infant sex, *n* (%)							
Boys	13,116,306	11,817,631 (51.2)	1,298,675 (51.2)	12,139,255 (51.2)	977,051 (51.3)	12,274,869 (51.2)	841,437 (51.3)
Girls	12,507,173	11,269,873 (48.8)	1,237,300 (48.8)	11,579,662 (48.8)	927,511 (48.7)	11,708,821 (48.8)	798,352 (48.7)
Preterm birth, *n* (%)							
Yes	2,378,398	2,066,183 (9.0)	312,215 (12.3)	2,122,518 (9.0)	255,880 (13.4)	2,151,292 (9.0)	227,106 (13.9)
No	2,324,5081	21,021,321 (91.1)	2,223,760 (87.7)	21,596,399 (91.1)	1,648,682 (86.6)	21,832,398 (91.0)	1,412,683 (86.2)

**Fig 2:**
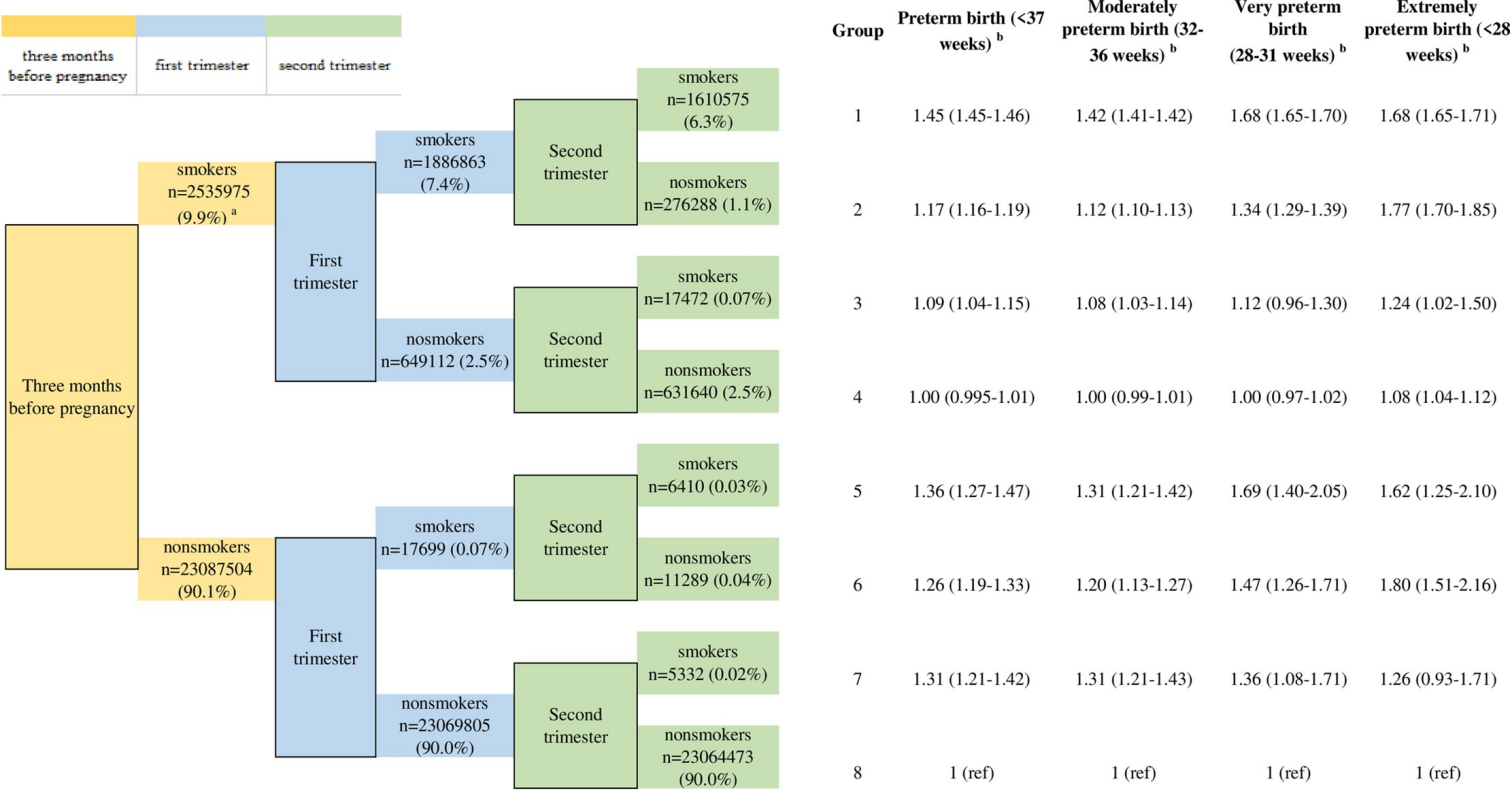
Association of the timing of maternal cigarette smoking with risk of preterm birth. ^a^Number of participants and its percentage among the overall population. ^b^The adjusted OR of preterm birth for women with different smoking patterns (groups 1–7) compared with nonsmokers (group 8). Adjustment for maternal age, race/ethnicity, parity, education levels, prepregnancy BMI, previous history of preterm birth, marital status, infant sex, and timing of initiation of prenatal care. OR, odds ratio; ref, reference.

Maternal smoking during either the first or second trimester of pregnancy was associated with an increased risk of preterm birth. After adjustment for maternal age, race/ethnicity, parity, education levels, previous history of preterm birth, marital status, prepregnancy BMI, infant sex, and timing of initiation of prenatal care, the OR of preterm birth compared with nonsmokers was 1.00 (95% CI 1.00–1.01) for mothers who only smoked before pregnancy (i.e., group 8), 1.26 (1.19–1.33) for those who only smoked during the first trimester (i.e., group 6), and 1.31 (1.21–1.42) for those who only smoked during the second trimester (i.e., group 7). For mothers who quit smoking during the first trimester (i.e., group 2) and those who smoked through the second trimester (i.e., group 1), the corresponding ORs were 1.17 (1.16–1.19) and 1.45 (1.45–1.46), respectively (all *P* values < 0.001). Positive and significant associations were observed for mothers who smoked in multiple trimesters, as well (**Fig 2**). Similar results were observed for a finer categorization of preterm birth (**Fig 2**).

In terms of smoking intensity, significant associations of preterm birth were observed for smoking cigarettes, even at low levels (1–2 cigarettes per day), at either 3 months before pregnancy or the first or second trimester (**Table 2**). The adjusted ORs of preterm delivery for mothers who smoked 0, 1–2, 3–5, 6–9, 10–19, and ≥20 cigarettes per day before pregnancy were 1.00 (reference group), 1.22 (1.20–1.24), 1.23 (1.22–1.24), 1.21 (1.19–1.23), 1.32 (1.31–1.33), and 1.36 (1.35–1.37), respectively (all *P* values < 0.001). For those who smoked during the first trimester, the corresponding ORs were 1.00 (reference group), 1.31 (1.29–1.33), 1.31 (1.30–1.32), 1.33 (1.31–1.35), 1.44 (1.43–1.45), and 1.53 (1.52–1.55), respectively (all *P* values < 0.001). For those who smoked during the second trimester, the corresponding ORs were 1.00 (reference group), 1.37 (1.35–1.39), 1.36 (1.35–1.38), 1.36 (1.34–1.38), 1.48 (1.47–1.49), and 1.59 (1.58–1.61), respectively (all *P* values < 0.001). Significant associations were observed for a finer categorization of preterm birth (**Table 2**).

**Table 2:** Associations of the intensity of maternal cigarette smoking before and during pregnancy with risk of preterm birth.


Cigarettes per day	No. of participants, *n* (%)	Preterm birth (<37 weeks)		Moderately preterm birth (32–36 weeks)		Very preterm birth (28–31 weeks)		Extremely preterm birth (<28 weeks)	
		No. of cases, *n* (%)	Adjusted OR (95% CI)	No. of cases, *n* (%)	Adjusted OR (95% CI)	No. of cases, *n* (%)	Adjusted OR (95% CI)	No. of cases, n (%)	Adjusted OR (95% CI)
**Before pregnancy**									
0	23,087,504 (90.1)	2,066,183 (8.1)	1.00 (ref)	1,760,014 (6.9)	1.00 (ref)	190,829 (0.7)	1.00 (ref)	115,340 (0.5)	1.00 (ref)
1–2	166,716 (0.7)	20,291 (0.1)	1.22 (1.20–1.24)	16,682 (0.1)	1.20 (1.18–1.22)	2,224 (0.01)	1.34 (1.29–1.40)	1,385 (0.01)	1.37 (1.30–1.45)
3–5	466,906 (1.8)	56,572 (0.2)	1.23 (1.22–1.24)	46,559 (0.2)	1.20 (1.19–1.21)	6,182 (0.02)	1.36 (1.33–1.40)	3,831 (0.01)	1.43 (1.38–1.47)
6–9	177,060 (0.7)	21,047 (0.1)	1.21 (1.19–1.23)	17,460 (0.1)	1.19 (1.17–1.21)	2,259 (0.01)	1.32 (1.27–1.38)	1,328 (0.01)	1.35 (1.28–1.43)
10–19	793,833 (3.1)	96,696 (0.4)	1.32 (1.31–1.33)	80,201 (0.3)	1.28 (1.27–1.29)	10,473 (0.04)	1.51 (1.48–1.54)	6,022 (0.02)	1.64 (1.60–1.69)
≥20	931,460 (3.6)	117,609 (0.5)	1.36 (1.35–1.37)	98,320 (0.4)	1.33 (1.32–1.34)	12,546 (0.05)	1.53 (1.50–1.56)	6,743 (0.03)	1.59 (1.54–1.63)
**First trimester**									
0	23,718,917 (92.6)	2,122,518 (8.3)	1.00 (ref)	1,807,803 (7.1)	1.00 (ref)	196,072 (0.8)	1.00 (ref)	118,643 (0.5)	1.00 (ref)
1–2	158,822 (0.6)	20,834 (0.1)	1.31 (1.29–1.33)	16,985 (0.1)	1.27 (1.25–1.29)	2,575 (0.01)	1.49 (1.43–1.55)	1,474 (0.01)	1.53 (1.45–1.61)
3–5	454,726 (1.8)	58,356 (0.2)	1.31 (1.30–1.32)	47,728 (0.2)	1.27 (1.26–1.28)	6,537 (0.03)	1.50 (1.46–1.54)	4,091 (0.02)	1.64 (1.59–1.70)
6–9	153,226 (0.6)	19,912 (0.1)	1.33 (1.31–1.35)	16,468 (0.1)	1.30 (1.28–1.32)	2,199 (0.01)	1.51 (1.45–1.58)	1,245 (0.004)	1.55 (1.46–1.64)
10–19	699,294 (2.7)	93,204 (0.4)	1.44 (1.43–1.45)	77,147 (0.3)	1.39 (1.38–1.40)	10,343 (0.04)	1.69 (1.66–1.73)	5,714 (0.02)	1.82 (1.77–1.88)
≥20	438,494 (1.7)	63,574 (0.2)	1.53 (1.52–1.55)	53,105 (0.2)	1.50 (1.48–1.51)	6,987 (0.03)	1.76 (1.71–1.80)	3,482 (0.01)	1.71 (1.65–1.77)
**Second trimester**									
0	23,983,690 (93.6)	2,151,292 (8.4)	1.00 (ref)	1,830,930 (7.1)	1.00 (ref)	199,281 (0.8)	1.00 (ref)	121,081 (0.5)	1.00 (ref)
1–2	150,886 (0.6)	20,925 (0.1)	1.37 (1.35–1.39)	17,088 (0.1)	1.33 (1.31–1.36)	2,400 (0.01)	1.56 (1.50–1.63)	1,437 (0.01)	1.56 (1.48–1.64)
3–5	460,484 (1.8)	61,371 (0.2)	1.36 (1.35–1.38)	50,372 (0.2)	1.32 (1.31–1.34)	6,971 (0.03)	1.58 (1.55–1.62)	4,028 (0.02)	1.62 (1.57–1.68)
6–9	152,266 (0.6)	20,077 (0.08)	1.36 (1.34–1.38)	16,788 (0.1)	1.34 (1.31–1.34)	2,155 (0.01)	1.51 (1.44–1.57)	1,134 (0.004)	1.46 (1.38–1.55)
10–19	617,587 (2.4)	84,897 (0.3)	1.48 (1.47–1.49)	70,978 (0.3)	1.44 (1.43–1.46)	9,243 (0.04)	1.70 (1.66–1.74)	4,676 (0.02)	1.69 (1.63–1.74)
≥20	258,566 (1.0)	39,836 (0.2)	1.59 (1.58–1.61)	33,080 (0.1)	1.55 (1.53–1.57)	4,463 (0.02)	1.84 (1.79–1.90)	2,293 (0.01)	1.80 (1.72–1.88)

Adjustment for maternal age, race/ethnicity, parity, education levels, prepregnancy BMI, previous history of preterm birth, marital status, infant sex, and timing of initiation of prenatal care.

Abbreviations: CI, confidence interval; NVSS, National Vital Statistics System; OR, odds ratio; ref, reference

It is worth noting that 74.4% of women who smoked before pregnancy continued to smoke during the first trimester, among which 85.4% smoked during the second trimester. Therefore, we examined the association of smoking cessation at various periods during pregnancy with preterm birth to further understand the effects of smoking intensity at various periods (**Table 3**). Smokers who quit before pregnancy, regardless of smoking intensity, had a comparable risk of preterm birth with nonsmokers. However, women who quit during pregnancy showed a significant increased risk of preterm birth, even for those who smoked only 1–2 cigarettes per day.

**Table 3:** Maternal smoking cessation at various periods and risk of preterm birth.

Cigarettes per day	Cessation before pregnancy	Cessation during the first trimester	Smoking throughout the second trimester
Never smoking	1.00 (ref)	1.00 (ref)	1.00 (ref)
**Before pregnancy**			
1–2	0.99 (0.97–1.02)*	1.23 (1.18–1.29)	1.46 (1.43–1.49)
3–5	0.99 (0.98–1.01)	1.16 (1.13–1.19)	1.43 (1.41–1.45)
6–9	0.97 (0.94–1.00)	1.14 (1.09–1.19)	1.36 (1.33–1.39)
10–19	1.00 (0.99–1.02)	1.15 (1.13–1.18)	1.46 (1.45–1.47)
≥20	1.01 (0.99–1.03)	1.18 (1.15–1.20)	1.46 (1.45–1.47)
**First trimester**			
1–2	NA	1.13 (1.10–1.16)	1.39 (1.36–1.41)
3–5	NA	1.13 (1.10–1.15)	1.36 (1.35–1.37)
6–9	NA	1.19 (1.14–1.25)	1.35 (1.33–1.37)
10–19	NA	1.19 (1.16–1.22)	1.47 (1.46–1.48)
≥20	NA	1.26 (1.22–1.30)	1.56 (1.54–1.57)
**Second trimester**			
1–2	NA	NA	1.38 (1.36–1.40)
3–5	NA	NA	1.37 (1.36–1.38)
6–9	NA	NA	1.37 (1.35–1.39)
10–19	NA	NA	1.49 (1.47–1.50)
≥20	NA	NA	1.60 (1.58–1.62)

Cessation before pregnancy: smoking before pregnancy, but no smoking during the first trimester and the second trimester.

Cessation during the first trimester: smoking before pregnancy and during the first trimester, but no smoking during the second trimester.

Smoking throughout the second trimester: smoking before pregnancy and during the first and second trimesters of pregnancy

Never smoking: no smoking before or during pregnancy.

Adjustment for maternal age, race/ethnicity, parity, education levels, prepregnancy BMI, previous history of preterm birth, marital status, infant sex, and initiation of prenatal care.

*Adjusted OR (95% CI).

Abbreviations: CI, confidence interval; NA, not applicable; OR, odds ratio; ref, reference

The association of smoking status and daily smoking amount with preterm birth was modified by age, race/ethnicity, and education levels (*P* for interaction values < 0.001). Although maternal smoking during pregnancy was associated with preterm birth in each age group, race/ethnicity group, and education levels (**S2 Table, S3 Table, S4 Table**), the associations were more prominent among women 35 years or older, non-Hispanic whites, and those with higher education levels. For the intensity of cigarette consumption, a significant association between cigarette consumption, even only 1–2 cigarettes per day, during pregnancy and preterm birth was observed consistently in age, race/ethnicity, and education groups (**S5 Table, S6 Table, S7 Table**), and the associations were more prominent among women ≥ 35 years old, non-Hispanic whites, and those with higher education levels.

In the sensitivity analysis using data from NVSS 2014–2018, we found that maternal smoking before pregnancy or during either the first or second trimester of pregnancy was associated with an increased risk of preterm birth (**S1 Fig**); meanwhile, smoking, even at low levels (1–2 cigarettes per day), before or during pregnancy was associated with a higher risk of preterm birth compared with nonsmoking (**S8 Table**). These were consistent with our main findings. In addition, similar results were observed in a propensity score analysis (**S9 Table, S10 Table, S11 Table**).

## Discussion

In the present study with data from more than 25 million mother–infant pairs, we found that maternal smoking during pregnancy, either the first or second trimester, was positively and significantly associated with preterm birth in a dose–response manner. Low-intensity smoking, even only 1–2 cigarettes per day, during the first or second trimester was associated with increased risk of preterm birth. Compared with nonsmokers, smokers who quit before pregnancy showed a comparable risk of preterm birth, but the risk increased for those who quit during pregnancy, even if only as few as 1–2 cigarettes were smoked daily. Our data highlight that no level is safe for cigarette smoking during pregnancy.

### Comparison with previous studies

This study provided a comprehensive view regarding the timing and intensity of maternal cigarette smoking and preterm birth. We found that maternal smoking during either the first or second trimester of pregnancy was associated with an increased risk of preterm birth. This is an important public health message because findings from previous studies in this regard have been inconsistent and inconclusive. Several previous studies showed that mothers who quit smoking during pregnancy had a similar risk of preterm birth to nonsmokers [13,30,31]. Disparities in sample size and population as well as confounders included in analyses may contribute to the inconsistent findings. For example, in a previous study, there were only 10 spontaneous preterm birth cases in the stopped-smoker group, which may restrict the statistical power to detect significant differences [13]. In another study, smoking cessation during pregnancy was not associated with preterm birth in a large cohort study [15]. However, quitters in that study were defined as women who smoked 3 months before pregnancy but stopped before 8–12 gestational weeks; therefore, they were unable to distinguish whether women quit smoking before pregnancy or during the first trimester. In addition, a more recent study using the same dataset as the one we used reported that smoking cessation was associated with reduced probabilities of preterm birth [23]. However, they did not examine the risk of preterm birth compared with nonsmokers, especially for finer categories of smoking levels. In addition, our study found that maternal smoking before pregnancy was associated with extremely preterm birth, although there was no association with moderately preterm birth or very preterm birth. This supports the recommendation that women who smoke should be advised to quit smoking before pregnancy.

Regarding the intensity of cigarette smoking, we found that mothers who smoked even just 1–2 cigarettes per day during either the first or the second trimester were at a higher risk of preterm delivery compared with nonsmokers. Previous studies usually define light smoking as smoking 1–5 or 1–9 cigarettes per day, and they have yielded inconsistent findings for the association of maternal light smoking with risk of preterm birth [12,16,20,21]. In the present study, we were able to apply a finer category of light smoking because of the extremely large sample size. Although the magnitude of smoking fewer than 10 cigarettes per day was similar, significant associations were observed for each finer category. More research is needed to confirm our findings. For moderate and heavy smoking, our findings were consistent with previous studies [15,16,18]. Our findings were consistent in different age and race/ethnicity groups. Moreover, we found similar findings across categories of preterm birth, i.e., moderately, very, and extremely preterm birth.

For the analysis of smoking intensity before pregnancy, we also observed an increased risk of preterm birth for each smoking dose, which may be attributed to the high proportion of continued smokers during pregnancy. When analyzing the association of maternal smoking (qualitative and quantitative) with preterm birth among women who quit at various periods, respectively, we found that women who quit before pregnancy had comparable risk of preterm birth with nonsmokers (women who never smoked before or during pregnancy). However, smokers who quit during pregnancy, even during the first trimester, had significantly increased risks of preterm birth. This may be because women who quit later were exposed to higher accumulated levels of tobacco.

### Biological plausibility

The association between maternal cigarette smoking and preterm birth is biologically plausible. First, maternal smoking could result in fetal hypoxia, which is a proposed mechanism of preterm birth, although the specific process is unclear [32]. Second, smoking has effects on maternal immunity and predisposes individuals to infection and/or inflammation [2,32], leading to an increased risk of preterm birth [33]. Third, maternal smoking could change placental function directly through regulating the expression of nicotinic acetylcholine receptor subunits [34], affecting the placental proteome [34], and impairing functions of placental macrophages [35]. Other mechanisms involved may include disrupting maternal endocrine function, affecting myometrial contraction, and changing oxytocin sensitivity [32,36]. Because the half-lives of the main compounds of cigarette smoke are short, it seems reasonable that women who quit earlier are affected less, as we found in this study. Mechanisms of the dose–response relation between daily amount of cigarette consumption and preterm birth remain to be elucidated.

### Strengths and limitations

The main strength of this study is the large sample size, which provided high statistical power to thoroughly analyze the association of the timing and the intensity of maternal smoking with preterm birth. In addition, we used a finer category of light smoking to examine the dose–response relationship between very low-intensity smoking (1–2 cigarettes per day) and preterm birth during specific periods. This study has several limitations. First, maternal smoking before and during pregnancy was self-reported, which may be subject to information bias. A previous study showed a high correlation (r = 0.70) between self-reported number of cigarettes and objectively measured biomarker (i.e., cotinine) at any given time point across women, indicating that self-reported variations in smoking during pregnancy may reasonably reflect fetal exposure in epidemiological studies [37]. Nonetheless, we are unable to confirm the validity of self-reported dosage when it comes to low-level exposure. In addition, smoking intensity patterns were assessed by the number of cigarettes smoked per day in this study. However, there was no information on natural variability in smoking behavior to account for a more-thorough smoking exposure [24,25]. Second, for maternal prepregnancy smoking, NVSS only collected data about smoking 3 months before pregnancy. We could not differentiate never smokers from those who did not smoke in the last 3 months prior to pregnancy. Third, we used the data of the LMP to estimate gestational age because the data about the obstetric estimate of gestation at delivery were only available since 2014 [28]. However, we conducted a sensitivity analysis using data from 2014 to 2018, and the results were similar. Fourth, women with preexisting hypertension or diabetes were excluded from the analyses, among whom the generalizability of our findings was limited. Moreover, our analyses were conducted among adults, which would restrict the extrapolation of the findings to adolescent pregnancy. Fifth, information on nondaily use of cigarettes is unavailable on birth certificates. Sixth, the NVSS birth data did not specify the reason for each case of preterm birth, which precludes us from doing analyses for each subtype of preterm birth (e.g., spontaneous preterm birth or medically indicated). Seventh, women who quit smoking in pregnancy might switch to other tobacco products, such as e-cigarettes. However, the absence of information in NVSS on e-cigarette use during pregnancy restricted our ability to distinguish its association with preterm birth. Finally, although we have adjusted for many potential confounders, we cannot rule out the possibility of residual confounding by unknown or unmeasured factors.

### Implications

Our findings are of considerable clinical and public health implications by highlighting that no level is safe for cigarette smoking during pregnancy. In contrast to some recent studies showing that smoking during early pregnancy (first or second trimester) [16,17] or at low levels [12,20,21] was not associated with increased risk of preterm birth, our data highlight that smoking cessation, rather than reduction, before pregnancy should be highly encouraged and supported for women at reproductive age. Increased efforts from various professional sources and policymakers are needed.

## Conclusion

In this large-scale population-based study, we found that maternal smoking, even only 1–2 cigarettes per day, during either the first or the second trimester was associated with increased risk of preterm birth. Our findings support the current recommendation for smoking cessation before pregnancy by highlighting that there is no safe level or safe period for maternal smoking during pregnancy. In the future, increased efforts should be made to help women of reproductive age who smoke stop smoking completely before pregnancy to prevent preterm delivery.

## Supporting information

S1 STROBE Checklist STROBE, Strengthening the Reporting of Observational Studies in Epidemiology. (DOC)

S1 Fig The association of trimester-specific smoking status with preterm birth: NVSS, 2014–2018. NVSS, National Vital Statistics System.
(TIF)

S1 Table Percentage of preterm birth according to population characteristics: The National Vital Statistics System, 2011–2018. (DOCX)

S2 Table The association of trimester-specific smoking status with preterm birth according to age groups. (DOCX)

S3 Table The association of trimester-specific smoking status with preterm birth according to race/ethnicity. (DOCX)

S4 Table The association of trimester-specific smoking status with preterm birth according to education levels. (DOCX)

S5 Table The association of daily cigarette consumption with preterm birth according to age groups. (DOCX)

S6 Table The association of daily cigarette consumption with preterm birth according to race/ethnicity. (DOCX)

S7 Table The association of daily cigarette consumption with preterm birth according to education levels. (DOCX)

S8 Table The associations of smoking with preterm birth: NVSS, 2014–2018. NVSS, National Vital Statistics System.
(DOCX)

S9 Table Sensitivity analysis for the associations of timing of maternal smoking with preterm birth after additional adjustment for a propensity score. (DOCX)

S10 Table Sensitivity analysis for the associations of maternal smoking with preterm birth after additional adjustment for a propensity score. (DOCX)

S11 Table Sensitivity analysis for the association between smoking cessation at various periods and preterm birth after additional adjustment for a propensity score. (DOCX)

## Abbreviations

CDC: Centers for Disease Control and Prevention
CI: confidence interval
LMP: last menstrual period
NCHS: National Center for Health Statistics
NVSS: National Vital Statistics System
OR: odds ratio
STROBE: Strengthening the Reporting of Observational Studies in Epidemiology

## References

[1] Liu L, Oza S, Hogan D, Perin J, Rudan I, Lawn JE, et al Global, regional, and national causes of child mortality in 2000–13, with projections to inform post-2015 priorities: an updated systematic analysis. Lancet. 2015;385(9966):430–40. 10.1016/S0140-6736(14)61698-6 . 25280870

[2] Goldenberg RL, Culhane JF, Iams JD, Romero R. Epidemiology and causes of preterm birth. Lancet. 2008;371(9606):75–84. 10.1016/S0140-6736(08)60074-4 . 18177778 PMC7134569

[3] Chawanpaiboon S, Vogel JP, Moller AB, Lumbiganon P, Petzold M, Hogan D, et al Global, regional, and national estimates of levels of preterm birth in 2014: a systematic review and modelling analysis. Lancet Glob health. 2019;7(1):e37–e46. 10.1016/S2214-109X(18)30451-0 . 30389451 PMC6293055

[4] Martin JA, Hamilton BE, Osterman MJK, Driscoll AK. Births: Final Data for 2018. National vital statistics reports: from the Centers for Disease Control and Prevention, National Center for Health Statistics, National Vital Statistics System. 2019;68(13):1–47 [cited 2020 Feb 2]. Available from: https://www.cdc.gov/nchs/data/nvsr/nvsr68/nvsr68_13-508.pdf. 32501202

[5] Institute of Medicine (US) Committee on Understanding Premature Birth and Assuring Healthy Outcomes; Behrman RE, Butler AS, editors. Preterm Birth: Causes, Consequences, and Prevention. Washington, DC: National Academies Press (US); 2007 [cited 2020 Feb 2]. Available from: https://www.ncbi.nlm.nih.gov/books/NBK11362/. 10.17226/11622 20669423

[6] Lee AC, Blencowe H, Lawn JE. Small babies, big numbers: global estimates of preterm birth. Lancet Glob health. 2019;7(1):e2–e3. 10.1016/S2214-109X(18)30484-4 . 30389450

[7] National Center for Chronic Disease Prevention and Health Promotion Office on Smoking and Health. The Health Consequences of Smoking-50 Years of Progress: A Report of the Surgeon General. Atlanta: Centers for Disease Control and Prevention (US); 2014 [cited 2020 Feb 2]. Available from: https://www.ncbi.nlm.nih.gov/books/NBK179276/.

[8] Scherman A, Tolosa JE, McEvoy C. Smoking cessation in pregnancy: a continuing challenge in the United States. Ther Adv Drug Saf. 2018;9(8):457–74. 10.1177/2042098618775366 . 30364850 PMC6199686

[9] Lange S, Probst C, Rehm J, Popova S. National, regional, and global prevalence of smoking during pregnancy in the general population: a systematic review and meta-analysis. Lancet Glob health. 2018;6(7):e769–e76. 10.1016/S2214-109X(18)30223-7 . 29859815

[10] Drake P, Driscoll AK, Mathews TJ. Cigarette Smoking During Pregnancy: United States, 2016 NCHS data brief. 2018;(305):1–8. . 29528282

[11] Amrock SM, Weitzman M. Adolescents' perceptions of light and intermittent smoking in the United States. Pediatrics. 2015;135(2):246–54. 10.1542/peds.2014-2502 . 25583910 PMC4306801

[12] Jaddoe VW, Troe EJ, Hofman A, Mackenbach JP, Moll HA, Steegers EA, et al Active and passive maternal smoking during pregnancy and the risks of low birthweight and preterm birth: the Generation R Study. Paediatr Perinat Epidemiol. 2008;22(2):162–71. 10.1111/j.1365-3016.2007.00916.x . 18298691

[13] McCowan LM, Dekker GA, Chan E, Stewart A, Chappell LC, Hunter M, et al Spontaneous preterm birth and small for gestational age infants in women who stop smoking early in pregnancy: prospective cohort study. BMJ. 2009;338:b1081 10.1136/bmj.b1081 . 19325177 PMC2661373

[14] Yan J, Groothuis PA. Timing of prenatal smoking cessation or reduction and infant birth weight: evidence from the United Kingdom Millennium Cohort Study. Matern Child Health J. 2015;19(3):447–58. 10.1007/s10995-014-1516-x . 24889113

[15] Dahlin S, Gunnerbeck A, Wikstrom AK, Cnattingius S, Edstedt Bonamy AK. Maternal tobacco use and extremely premature birth—a population-based cohort study. BJOG. 2016;123(12):1938–46. 10.1111/1471-0528.14213 . 27411948

[16] Tong VT, England LJ, Rockhill KM, D'Angelo DV. Risks of Preterm Delivery and Small for Gestational Age Infants: Effects of Nondaily and Low-Intensity Daily Smoking During Pregnancy. Paediatr Perinat Epidemiol. 2017;31(2):144–8. 10.1111/ppe.12343 . 28181676 PMC6368675

[17] Moore E, Blatt K, Chen A, Van Hook J, DeFranco EA. Relationship of trimester-specific smoking patterns and risk of preterm birth. Am J Obstet Gynecol. 2016;215(1):109e1-6. 10.1016/j.ajog.2016.01.167 . 26827877 PMC5344039

[18] Ko TJ, Tsai LY, Chu LC, Yeh SJ, Leung C, Chen CY, et al Parental smoking during pregnancy and its association with low birth weight, small for gestational age, and preterm birth offspring: a birth cohort study. Pediatr Neonatol. 2014;55(1):20–7. 10.1016/j.pedneo.2013.05.005 . 23850094

[19] Shah NR, Bracken MB. A systematic review and meta-analysis of prospective studies on the association between maternal cigarette smoking and preterm delivery. Am J Obstet Gynecol. 2000;182(2):465–72. 10.1016/s0002-9378(00)70240-7 . 10694353 PMC2706697

[20] Berkowitz GS, Holford TR, Berkowitz RL. Effects of cigarette smoking, alcohol, coffee and tea consumption on preterm delivery. Early Hum Dev. 1982;7(3):239–50. 10.1016/0378-3782(82)90086-x . 7160334

[21] Haskins A, Mukhopadhyay S, Pekow P, Markenson G, Bertone-Johnson E, Carbone E, et al Smoking and risk of preterm birth among predominantly Puerto Rican women. Ann Epidemiol. 2008;18(6):440–6. 10.1016/j.annepidem.2008.02.002 . 18538266

[22] Martin JA, Hamilton BE, Osterman MJK, Driscoll AK, Drake P. Births: Final Data for 2017. National vital statistics reports: from the Centers for Disease Control and Prevention, National Center for Health Statistics, Natl Vital Stat Rep. 2018;67(8):1–50 [cited 2020 Feb 2]. Available from: https://www.cdc.gov/nchs/data/nvsr/nvsr67/nvsr67_08-508.pdf. 30707672

[23] Soneji S, Beltran-Sanchez H. Association of Maternal Cigarette Smoking and Smoking Cessation With Preterm Birth. JAMA Netw Open. 2019;2(4):e192514 10.1001/jamanetworkopen.2019.2514 31002320 PMC6481448

[24] Kondracki AJ, Hofferth SL. A gestational vulnerability window for smoking exposure and the increased risk of preterm birth: how timing and intensity of maternal smoking matter. Reprod Health. 2019;16(1):43 10.1186/s12978-019-0705-x . 30992027 PMC6469085

[25] Kondracki AJ. Prevalence and patterns of cigarette smoking before and during early and late pregnancy according to maternal characteristics: the first national data based on the 2003 birth certificate revision, United States, 2016. Reprod Health. 2019;16(1):142 10.1186/s12978-019-0807-5 . 31519184 PMC6743116

[26] Martin JA. United States vital statistics and the measurement of gestational age. Paediatr Perinat Epidemiol. 2007;21 Suppl 2:13–21. 10.1111/j.1365-3016.2007.00857.x . 17803614

[27] Tucker J, McGuire W. Epidemiology of preterm birth. BMJ. 2004;329(7467):675–8. 10.1136/bmj.329.7467.675 . 15374920 PMC517653

[28] Martin JA, Osterman MJ, Kirmeyer SE, Gregory EC. Measuring Gestational Age in Vital Statistics Data: Transitioning to the Obstetric Estimate. Natl Vital Stat Rep. 2015;64(5):1–20 [cited 2020 Feb 2]. Available from: https://www.cdc.gov/nchs/data/nvsr/nvsr64/nvsr64_05.pdf. 26047089

[29] D'Agostino RB Jr. Propensity score methods for bias reduction in the comparison of a treatment to a non-randomized control group. Stat Med. 1998;17(19):2265–81. 10.1002/(sici)1097-0258(19981015)17:19&lt;2265::aid-sim918&gt;3.0.co;2-b . 9802183

[30] Cnattingius S, Granath F, Petersson G, Harlow BL. The influence of gestational age and smoking habits on the risk of subsequent preterm deliveries. N Engl J Med. 1999;341(13):943–8. 10.1056/NEJM199909233411303 . 10498489

[31] Li CQ, Windsor RA, Perkins L, Goldenberg RL, Lowe JB. The impact on infant birth weight and gestational age of cotinine-validated smoking reduction during pregnancy. JAMA. 1993;269(12):1519–24. 10.1001/jama.1993.03500120057026 . 8445814

[32] Ion R, Bernal AL. Smoking and Preterm Birth. Reprod Sci. 2015;22(8):918–26. 10.1177/1933719114556486 . 25394641

[33] Romero R, Espinoza J, Goncalves LF, Kusanovic JP, Friel L, Hassan S. The role of inflammation and infection in preterm birth. Semin Reprod Med. 2007;25(1):21–39. 10.1055/s-2006-956773 . 17205421 PMC8324073

[34] Machaalani R, Ghazavi E, Hinton T, Waters KA, Hennessy A. Cigarette smoking during pregnancy regulates the expression of specific nicotinic acetylcholine receptor (nAChR) subunits in the human placenta. Toxicol Appl Pharmacol. 2014;276(3):204–12. 10.1016/j.taap.2014.02.015 . 24607864

[35] Belhareth R, Mezouar S, Ben Amara A, Chartier C, Azzouz EB, Chabriere E, et al Cigarette smoke extract interferes with placenta macrophage functions: A new mechanism to compromise placenta functions? Reprod Toxicol. 2018;78:120–9. 10.1016/j.reprotox.2018.04.009 . 29673796

[36] Egawa M, Yasuda K, Nakajima T, Okada H, Yoshimura T, Yuri T, et al Smoking enhances oxytocin-induced rhythmic myometrial contraction. Biol Reprod. 2003;68(6):2274–80. 10.1095/biolreprod.102.010785 . 12606462

[37] Pickett KE, Rathouz PJ, Kasza K, Wakschlag LS, Wright R. Self-reported smoking, cotinine levels, and patterns of smoking in pregnancy. Paediatr Perinat Epidemiol. 2005;19(5):368–76. 10.1111/j.1365-3016.2005.00660.x . 16115289

