# Supplementary figures and images for: Maternal cigarette smoking before and during pregnancy and the risk of preterm birth: A dose–response analysis of 25 million mother–infant pairs
Source: PLoS Med. 2020 Aug 18;17(8):e1003158. doi: 10.1371/journal.pmed.1003158 (PMC7446793; doi:10.1371/journal.pmed.1003158)

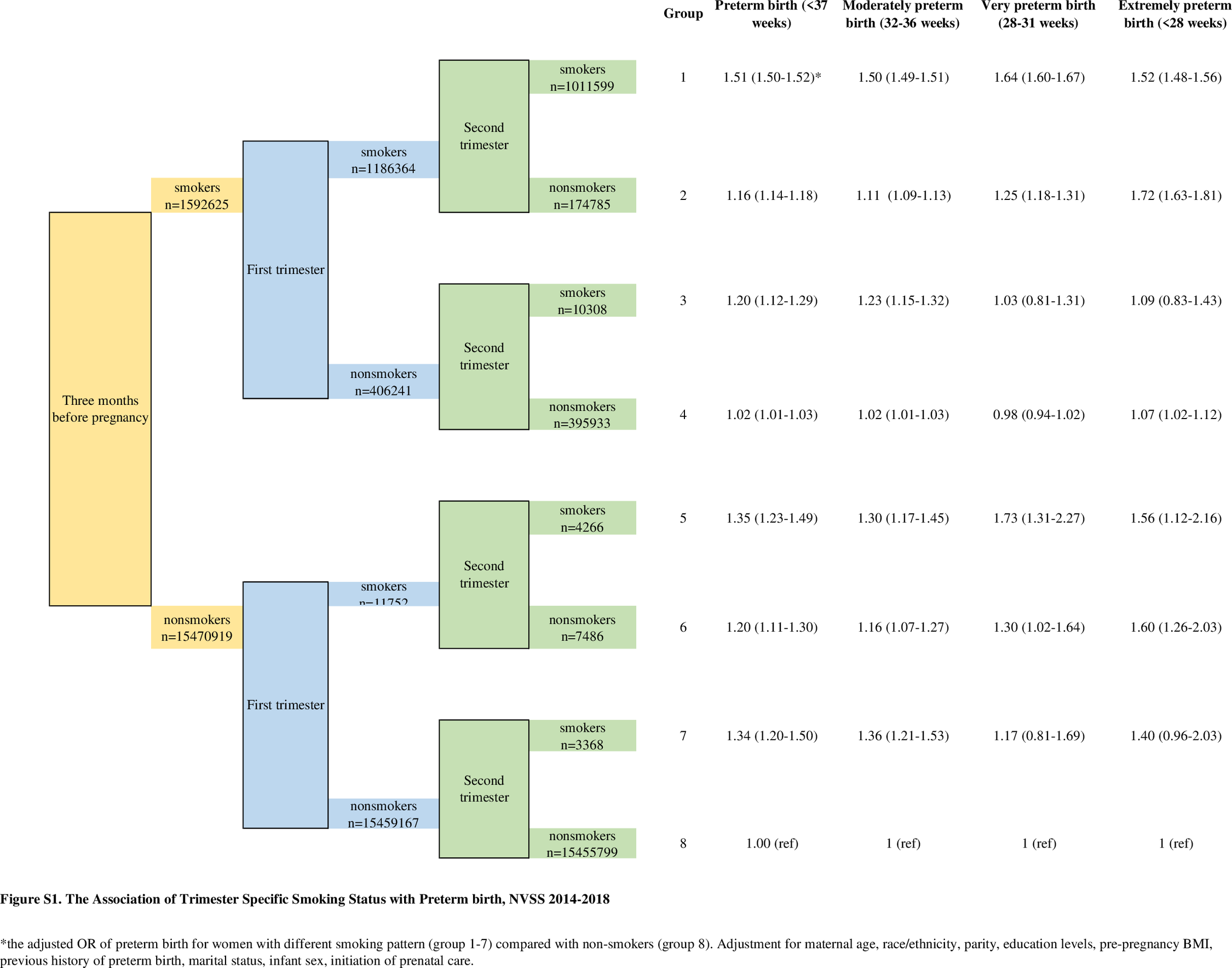

Supplement: S1 Fig — NVSS, National Vital Statistics System. (TIF) [file pmed.1003158.s002.tif]
