# Supplementary material for: Maternal cigarette smoking before and during pregnancy and the risk of preterm birth: A dose–response analysis of 25 million mother–infant pairs
Source: PLoS Med. 2020 Aug 18;17(8):e1003158. doi: 10.1371/journal.pmed.1003158 (PMC7446793; doi:10.1371/journal.pmed.1003158)
Supplement: S1 Table — (DOCX) [file pmed.1003158.s003.docx]

**S1 Table. Percentage of Preterm Birth according to Population Characteristics: The National Vital Statistics System 2011–2018**

| **Variables** | **No. of participants** | **Preterm birth, *n* (%)** | **Moderately preterm birth, *n* (%)** | **Very preterm birth, *n* (%)** | **Extremely preterm birth, *n* (%)** |
| --- | --- | --- | --- | --- | --- |
| Overall | 25,623,479 | 2,378,398 (9.3) | 2,019,236 (7.9) | 224,513 (0.9) | 134,649 (0.5) |
| Age, years |  |  |  |  |  |
| 20-24 | 5,981,701 | 598,768 (10.0) | 502,735 (8.4) | 59,903 (1.0) | 36,130 (0.6) |
| 25-29 | 7,958,816 | 694,819 (8.7) | 590,422 (7.4) | 64,868 (0.8) | 39,529 (0.5) |
| 30-34 | 7,383,822 | 631,394 (8.6) | 539,990 (7.3) | 57,347 (0.8) | 34,057 (0.5) |
| 35-39 | 3,511,546 | 352,846 (10.1) | 300,777 (8.6) | 32,634 (0.9) | 19,435 (0.6) |
| ≥40 | 787,594 | 100,571 (12.8) | 85,312 (10.8) | 9,761 (1.2) | 5,498 (0.7) |
| Race/ethnicity |  |  |  |  |  |
| Hispanic | 5,971,598 | 584,939 (9.8) | 502,633 (8.4) | 51,945 (0.9) | 30,361 (0.5) |
| non-Hispanic white | 13,742,486 | 1,098,338 (8.0) | 951,479 (6.9) | 98,234 (0.7) | 48,625 (0.4) |
| non-Hispanic black | 3,417,456 | 476,885 (14.0) | 377,516 (11.1) | 55,007 (1.6) | 44,362 (1.3) |
| Other | 2,491,939 | 218,236 (8.8) | 187,608 (7.5) | 19,327 (0.8) | 11,301 (0.5) |
| Education levels | | | | | |
| Lower than high school | 3,218,469 | 386,542 (12.0) | 327,316 (10.2) | 38,324 (1.2) | 20,902 (0.7) |
| High school | 6,147,452 | 661,350 (10.8) | 554,581 (9.0) | 65,677 (1.1) | 41,092 (0.7) |
| Higher than high school | 15,960,302 | 1,299,315 (8.1) | 1,113,444 (7.0) | 117,347 (0.7) | 68,524 (0.4) |
| Missing | 297,256 | 31,191 (10.5) | 23,895 (8.0) | 3,165 (1.1) | 4,131 (1.4) |
| Marital status | | | | | |
| Married | 10,196,846 | 813,962 (8.0) | 705,556 (6.9) | 69,916 (0.7) | 38,490 (0.4) |
| Unmarried | 6,022,000 | 711,371 (11.8) | 587,914 (9.8) | 75,688 (1.3) | 47,769 (0.8) |
| Missing | 9,404,633 | 853,065 (9.1) | 725,766 (7.7) | 78,909 (0.8) | 48,390 (0.5) |
| Parity |  |  |  |  |  |
| 1 | 9,292,065 | 821,304 (8.8) | 677,856 (7.3) | 83,919 (0.9) | 59,529 (0.6) |
| 2 | 8,451,200 | 699,401 (8.3) | 604,535 (7.2) | 61,575 (0.7) | 33,291 (0.4) |
| 3 | 4,524,658 | 441,563 (9.8) | 381,676 (8.4) | 39,540 (0.9) | 20,347 (0.5) |
| ≥4 | 3,258,029 | 405,351 (12.4) | 346,681 (10.6) | 38,309 (1.2) | 20,361 (0.6) |
| Missing | 97,527 | 10,779 (11.1) | 8,488 (8.7) | 1,170 (1.2) | 1,121 (1.2) |
| Initiation of prenatal care | | | | | |
| No prenatal care | 36,7407 | 86,342 (23.5) | 62,385 (17.0) | 11,730 (3.2) | 12,227 (3.3) |
| 1st to 3rd month | 19,172,780 | 1,635,281 (8.5) | 1,406,306 (7.3) | 142,578 (0.7) | 86,397 (0.5) |
| 4th to 6th month | 4,266,280 | 452,516 (10.6) | 382,113 (9.0) | 48,191 (1.1) | 22,212 (0.5) |
| 7th to final month | 1,084,939 | 100,389 (9.3) | 89,745 (8.3) | 9,060 (0.8) | 1,584 (0.2) |
| Missing | 732,073 | 103,870 (14.2) | 78,687 (10.8) | 12,954 (1.8) | 12,229 (1.7) |
| Previous history of preterm birth |  |  |  |  |  |
| Yes | 729,606 | 188,382 (7.8) | 154,270 (21.1) | 20,765 (2.9) | 13,347 (1.8) |
| No | 15,581,179 | 1,365,906 (8.8) | 1,184,941 (7.6) | 119,507 (0.8) | 61,458 (0.4) |
| Nulliparous | 9,281,261 | 819,613 (8.8) | 676,699 (7.3) | 83,682 (0.9) | 59,232 (0.6) |
| Missing | 31,433 | 4,497 (14.3) | 3,326 (10.6) | 559 (1.8) | 612 (2.0) |
| Prepregnancy BMI, kg/m^2^ |  |  |  |  |  |
| <18.5 | 858,637 | 95,038 (11.1) | 81,198 (9.5) | 9,104 (1.1) | 4,736 (0.6) |
| 18.5-24.9 | 11,282,192 | 960,950 (8.5) | 829,863 (7.4) | 86,493 (0.8) | 44,594 (0.4) |
| 25-29.9 | 6,500,909 | 591,039 (9.1) | 503,980 (7.8) | 54,995 (0.9) | 32,064 (0.5) |
| 30-34.9 | 3,476,089 | 344,043 (9.9) | 288,813 (8.3) | 33,406 (1.0) | 21,824 (0.6) |
| 35-39.9 | 1,640,155 | 169,946 (10.4) | 140,805 (8.6) | 16,997 (1.0) | 12,144 (0.7) |
| ≥40 | 1,100,926 | 122,010 (11.1) | 100,181 (9.1) | 12,455 (1.1) | 9,374 (0.9) |
| Missing | 764,571 | 95,372 (12.5) | 74,396 (9.7) | 11,063 (1.5) | 9,913 (1.3) |
| Infant sex |  |  |  |  |  |
| Boys | 13,116,306 | 1,281,835 (9.8) | 1,089,909 (8.3) | 120,201 (0.9) | 71,725 (0.6) |
| Girls | 12,507,173 | 1,096,563 (8.8) | 929,327 (7.4) | 104,312 (0.8) | 62,924 (0.5) |
| Smoking before pregnancy | | | | | |
| Yes | 2,535,975 | 312,215 (12.3) | 259,222 (10.2) | 33,684 (1.3) | 19,309 (0.8) |
| No | 23,087,504 | 2,066,183 (9.0) | 1,760,014 (7.6) | 190,829 (0.8) | 115,340 (0.5) |
| Smoking during the first trimester | | | | | |
| Yes | 1,904,562 | 255,880 (13.4) | 211,433 (11.1) | 28,441 (1.5) | 16,006 (0.8) |
| No | 23,718,917 | 2,122,518 (9.0) | 1,807,803 (7.6) | 196,072 (0.8) | 118,643 (0.5) |
| Smoking during the second trimester | | | | | |
| Yes | 1,639,789 | 227,106 (13.9) | 188,306 (11.5) | 25,232 (1.5) | 13,568 (0.8) |
| No | 23,983,690 | 2,151,292 (9.0) | 1,830,930 (7.6) | 199,281 (0.8) | 121,081 (0.5) |
