# Supplementary material for: Maternal cigarette smoking before and during pregnancy and the risk of preterm birth: A dose–response analysis of 25 million mother–infant pairs
Source: PLoS Med. 2020 Aug 18;17(8):e1003158. doi: 10.1371/journal.pmed.1003158 (PMC7446793; doi:10.1371/journal.pmed.1003158)
Supplement: S2 Table — (DOCX) [file pmed.1003158.s004.docx]

**S2 Table. The Association of Trimester-Specific Smoking Status with Preterm Birth According to Age Groups.**

| **Age** | **Before pregnancy** | **First trimester** | **Second trimester** | **Group** | **Adjusted OR (95%CI)** |
| --- | --- | --- | --- | --- | --- |
| **20-34 years** | Yes | Yes | Yes | 1 | 1.41 (1.40-1.42)* |
|  | Yes | Yes | No | 2 | 1.14 (1.13-1.16) |
|  | Yes | No | Yes | 3 | 1.07 (1.01-1.12) |
|  | Yes | No | No | 4 | 0.99 (0.98-1.00) |
|  | No | Yes | Yes | 5 | 1.34 (1.24-1.45) |
|  | No | Yes | No | 6 | 1.24 (1.17-1.32) |
|  | No | No | Yes | 7 | 1.29 (1.18-1.41) |
|  | No | No | No | 8 | 1.00 (ref) |
| **≥35 years** | Yes | Yes | Yes | 1 | 1.66 (1.64-1.69) |
|  | Yes | Yes | No | 2 | 1.38 (1.33-1.44) |
|  | Yes | No | Yes | 3 | 1.27 (1.07-1.50) |
|  | Yes | No | No | 4 | 1.12 (1.09-1.15) |
|  | No | Yes | Yes | 5 | 1.42 (1.15-1.75) |
|  | No | Yes | No | 6 | 1.35 (1.15-1.59) |
|  | No | No | Yes | 7 | 1.40 (1.11-1.75) |
|  | No | No | No | 8 | 1.00 (ref) |

Adjustment for race/ethnicity, parity, education levels, pre-pregnancy BMI, previous history of preterm birth, marital status, infant sex, initiation of prenatal care.

Yes means smoking; No means not smoking.
