# Supplementary material for: Maternal cigarette smoking before and during pregnancy and the risk of preterm birth: A dose–response analysis of 25 million mother–infant pairs
Source: PLoS Med. 2020 Aug 18;17(8):e1003158. doi: 10.1371/journal.pmed.1003158 (PMC7446793; doi:10.1371/journal.pmed.1003158)
Supplement: S3 Table — (DOCX) [file pmed.1003158.s005.docx]

**S3 Table. The Association of Trimester-Specific Smoking Status with Preterm Birth According to Race/Ethnicity.**

| **Race/ethnicity** | **Before pregnancy** | **First trimester** | **Second trimester** | **Group** | **Adjusted OR (95%CI)** |
| --- | --- | --- | --- | --- | --- |
| **Hispanic** | Yes | Yes | Yes | 1 | 1.41 (1.39-1.44) |
|  | Yes | Yes | No | 2 | 1.15 (1.11-1.20) |
|  | Yes | No | Yes | 3 | 1.10 (0.91-1.33) |
|  | Yes | No | No | 4 | 0.98 (0.95-1.001) |
|  | No | Yes | Yes | 5 | 1.45 (1.14-1.84) |
|  | No | Yes | No | 6 | 1.40 (1.22-1.61) |
|  | No | No | Yes | 7 | 1.44 (1.14-1.82) |
|  | No | No | No | 8 | 1.00 (ref) |
| **Non-Hispanic white** | Yes | Yes | Yes | 1 | 1.47 (1.46-1.48) |
|  | Yes | Yes | No | 2 | 1.17 (1.15-1.19) |
|  | Yes | No | Yes | 3 | 1.11 (1.04-1.18) |
|  | Yes | No | No | 4 | 1.00 (0.99-1.01) |
|  | No | Yes | Yes | 5 | 1.41 (1.29-1.55) |
|  | No | Yes | No | 6 | 1.25 (1.15-1.36) |
|  | No | No | Yes | 7 | 1.37 (1.23-1.54) |
|  | No | No | No | 8 | 1.00 (ref) |
| **Non-Hispanic black** | Yes | Yes | Yes | 1 | 1.27 (1.25-1.29) |
|  | Yes | Yes | No | 2 | 1.12 (1.09-1.15) |
|  | Yes | No | Yes | 3 | 1.00 (0.90-1.13) |
|  | Yes | No | No | 4 | 0.99 (0.97-1.01) |
|  | No | Yes | Yes | 5 | 1.12 (0.96-1.30) |
|  | No | Yes | No | 6 | 1.16 (1.04-1.29) |
|  | No | No | Yes | 7 | 1.15 (0.99-1.33) |
|  | No | No | No | 8 | 1.00 (ref) |
| **Others** | Yes | Yes | Yes | 1 | 1.32 (1.30-1.35) |
|  | Yes | Yes | No | 2 | 1.13 (1.07-1.18) |
|  | Yes | No | Yes | 3 | 0.91 (0.74-1.12) |
|  | Yes | No | No | 4 | 1.01 (0.97-1.05) |
|  | No | Yes | Yes | 5 | 1.47 (1.15-1.90) |
|  | No | Yes | No | 6 | 1.23 (1.01-1.48) |
|  | No | No | Yes | 7 | 1.20 (0.90-1.60) |
|  | No | No | No | 8 | 1.00 (ref) |

Adjustment for maternal age, parity, education levels, pre-pregnancy BMI, previous history of preterm birth, marital status, infant sex, initiation of prenatal care.

Yes means smoking; No means not smoking**.**
