# Supplementary material for: Maternal cigarette smoking before and during pregnancy and the risk of preterm birth: A dose–response analysis of 25 million mother–infant pairs
Source: PLoS Med. 2020 Aug 18;17(8):e1003158. doi: 10.1371/journal.pmed.1003158 (PMC7446793; doi:10.1371/journal.pmed.1003158)
Supplement: S4 Table — (DOCX) [file pmed.1003158.s006.docx]

**S4 Table. The Association of Trimester-Specific Smoking Status with Preterm Birth According to Education Levels.**

| **Race/ethnicity** | **Before pregnancy** | **First trimester** | **Second trimester** | **Adjusted OR (95%CI)** |
| --- | --- | --- | --- | --- |
| **Lower than high school** | Yes | Yes | Yes | 1.40 (1.39-1.42) |
|  | Yes | Yes | No | 1.14 (1.11-1.18) |
|  | Yes | No | Yes | 0.97 (0.87-1.08) |
|  | Yes | No | No | 1.02 (0.99-1.04) |
|  | No | Yes | Yes | 1.20 (1.03-1.39) |
|  | No | Yes | No | 1.22 (1.09-1.37) |
|  | No | No | Yes | 1.30 (1.12-1.51) |
|  | No | No | No | 1.00 (ref) |
| **High school** | Yes | Yes | Yes | 1.32 (1.31-1.34) |
|  | Yes | Yes | No | 1.06 (1.04-1.08) |
|  | Yes | No | Yes | 1.02 (0.94-1.10) |
|  | Yes | No | No | 0.92 (0.91-0.93) |
|  | No | Yes | Yes | 1.20 (1.06-1.35) |
|  | No | Yes | No | 1.23 (1.12-1.34) |
|  | No | No | Yes | 1.20 (1.05-1.36) |
|  | No | No | No | 1.00 (ref) |
| **Higher than high school** | Yes | Yes | Yes | 1.57 (1.55-1.58) |
|  | Yes | Yes | No | 1.25 (1.23-1.28) |
|  | Yes | No | Yes | 1.20 (1.11-1.30) |
|  | Yes | No | No | 1.04 (1.03-1.06) |
|  | No | Yes | Yes | 1.64 (1.46-1.85) |
|  | No | Yes | No | 1.30 (1.18-1.43) |
|  | No | No | Yes | 1.44 (1.25-1.65) |
|  | No | No | No | 1.00 (ref) |

Adjustment for maternal age race/ethnicity, parity, prepregnancy BMI, previous history of preterm birth, marital status, infant sex, initiation of prenatal care.

Yes indicates smoking; No indicates not smoking.
