# Supplementary material for: Maternal cigarette smoking before and during pregnancy and the risk of preterm birth: A dose–response analysis of 25 million mother–infant pairs
Source: PLoS Med. 2020 Aug 18;17(8):e1003158. doi: 10.1371/journal.pmed.1003158 (PMC7446793; doi:10.1371/journal.pmed.1003158)
Supplement: S5 Table — (DOCX) [file pmed.1003158.s007.docx]

**S5 Table. The Association of Daily Cigarette Consumption with Preterm Birth According to Age Groups.**

| **Cigarette per day** | **Adjusted OR (95% CI)** | | |
| --- | --- | --- | --- |
|  | **Before pregnancy** | **First trimester** | **Second trimester** |
| **20-34 years** | | | |
| 0 | 1.00 (ref) | 1.00 (ref) | 1.00 (ref) |
| 1-2 | 1.20 (1.18-1.22) | 1.28 (1.26-1.30) | 1.34 (1.32-1.36) |
| 3-5 | 1.21 (1.19-1.22) | 1.28 (1.27-1.29) | 1.33 (1.32-1.35) |
| 6-9 | 1.18 (1.17-1.20) | 1.30 (1.28-1.32) | 1.33 (1.31-1.35) |
| 10-19 | 1.29 (1.28-1.30) | 1.40 (1.39-1.41) | 1.44 (1.42-1.45) |
| ≥20 | 1.32 (1.31-1.33) | 1.48 (1.47-1.50) | 1.55 (1.53-1.57) |
| **≥35 years** | | | |
| 0 | 1.00 (ref) | 1.00 (ref) | 1.00 (ref) |
| 1-2 | 1.34 (1.29-1.41) | 1.50 (1.43-1.57) | 1.53 (1.46-1.60) |
| 3-5 | 1.36 (1.33-1.40) | 1.48 (1.44-1.52) | 1.55 (1.50-1.59) |
| 6-9 | 1.38 (1.32-1.44) | 1.50 (1.43-1.57) | 1.51 (1.44-1.58) |
| 10-19 | 1.49 (1.46-1.52) | 1.64 (1.61-1.68) | 1.71 (1.67-1.75) |
| ≥20 | 1.61 (1.58-1.65) | 1.78 (1.74-1.83) | 1.78 (1.73-1.84) |

Adjustment for race/ethnicity, parity, education levels, prepregnancy BMI, previous history of preterm birth, marital status, infant sex, initiation of prenatal care.
