# Supplementary material for: Maternal cigarette smoking before and during pregnancy and the risk of preterm birth: A dose–response analysis of 25 million mother–infant pairs
Source: PLoS Med. 2020 Aug 18;17(8):e1003158. doi: 10.1371/journal.pmed.1003158 (PMC7446793; doi:10.1371/journal.pmed.1003158)
Supplement: S6 Table — (DOCX) [file pmed.1003158.s008.docx]

**S6 Table. The Association of Daily Cigarette Consumption with Preterm Birth According to Race/Ethnicity.**

| **Cigarette per day** | **Adjusted OR (95% CI)** | | |
| --- | --- | --- | --- |
|  | **Before pregnancy** | **First trimester** | **Second trimester** |
| **Hispanic** | | | |
| 0 | 1.00 (ref) | 1.00 (ref) | 1.00 (ref) |
| 1-2 | 1.14 (1.09-1.18) | 1.31 (1.26-1.37) | 1.39 (1.33-1.46) |
| 3-5 | 1.19 (1.16-1.22) | 1.33 (1.29-1.37) | 1.37 (1.33-1.42) |
| 6-9 | 1.17 (1.11-1.23) | 1.25 (1.18-1.33) | 1.26 (1.18-1.34) |
| 10-19 | 1.21 (1.17-1.25) | 1.35 (1.31-1.40) | 1.45 (1.39-1.51) |
| ≥20 | 1.28 (1.24-1.32) | 1.51 (1.45-1.58) | 1.61 (1.52-1.70) |
| **Non-Hispanic white** | | | |
| 0 | 1.00 (ref) | 1.00 (ref) | 1.00 (ref) |
| 1-2 | 1.25 (1.22-1.28) | 1.31 (1.28-1.34) | 1.38 (1.35-1.41) |
| 3-5 | 1.25 (1.23-1.26) | 1.32 (1.30-1.33) | 1.38 (1.36-1.39) |
| 6-9 | 1.23 (1.20-1.25) | 1.36 (1.33-1.39) | 1.39 (1.36-1.41) |
| 10-19 | 1.33 (1.31-1.34) | 1.44 (1.43-1.46) | 1.48 (1.47-1.49) |
| ≥20 | 1.36 (1.35-1.37) | 1.54 (1.52-1.55) | 1.59 (1.57-1.61) |
| **Non-Hispanic black** | | | |
| 0 | 1.00 (ref) | 1.00 (ref) | 1.00 (ref) |
| 1-2 | 1.17 (1.14-1.21) | 1.24 (1.20-1.27) | 1.27 (1.24-1.31) |
| 3-5 | 1.16 (1.14-1.18) | 1.22 (1.20-1.24) | 1.24 (1.22-1.26) |
| 6-9 | 1.14 (1.11-1.18) | 1.21 (1.17-1.25) | 1.20 (1.16-1.25) |
| 10-19 | 1.19 (1.17-1.22) | 1.26 (1.23-1.29) | 1.28 (1.25-1.31) |
| ≥20 | 1.19 (1.17-1.22) | 1.29 (1.25-1.32) | 1.35 (1.30-1.39) |
| **Other** | | | |
| 0 | 1.00 (ref) | 1.00 (ref) | 1.00 (ref) |
| 1-2 | 1.20 (1.14-1.26) | 1.26 (1.19-1.33) | 1.31 (1.24-1.39) |
| 3-5 | 1.17 (1.13-1.21) | 1.25 (1.21-1.29) | 1.26 (1.22-1.31) |
| 6-9 | 1.13 (1.07-1.20) | 1.20 (1.13-1.28) | 1.25 (1.18-1.33) |
| 10-19 | 1.22 (1.19-1.26) | 1.31 (1.27-1.36) | 1.34 (1.29-1.39) |
| ≥20 | 1.26 (1.23-1.30) | 1.39 (1.33-1.45) | 1.44 (1.36-1.52) |

Adjustment for maternal age, parity, education levels, pre-pregnancy BMI, previous history of preterm birth, marital status, infant sex, initiation of prenatal care.
