# Supplementary material for: Maternal cigarette smoking before and during pregnancy and the risk of preterm birth: A dose–response analysis of 25 million mother–infant pairs
Source: PLoS Med. 2020 Aug 18;17(8):e1003158. doi: 10.1371/journal.pmed.1003158 (PMC7446793; doi:10.1371/journal.pmed.1003158)
Supplement: S7 Table — (DOCX) [file pmed.1003158.s009.docx]

**S7 Table. The Association of Daily Cigarette Consumption with Preterm Birth According to Education Levels.**

| **Cigarette per day** | **Adjusted OR (95% CI)** | | |
| --- | --- | --- | --- |
|  | **Before pregnancy** | **First trimester** | **Second trimester** |
| **Lower than high school** | | | |
| 0 | 1.00 (ref) | 1.00 (ref) | 1.00 (ref) |
| 1-2 | 1.28 (1.24-1.32) | 1.30 (1.26-1.34) | 1.33 (1.29-1.37) |
| 3-5 | 1.27 (1.25-1.30) | 1.30 (1.28-1.33) | 1.32 (1.29-1.34) |
| 6-9 | 1.23 (1.19-1.27) | 1.30 (1.26-1.34) | 1.33 (1.29-1.37) |
| 10-19 | 1.35 (1.33-1.37) | 1.41 (1.38-1.43) | 1.42 (1.40-1.44) |
| ≥20 | 1.35 (1.33-1.37) | 1.46 (1.44-1.49) | 1.53 (1.50-1.56) |
| **High school** | | | |
| 0 | 1.00 (ref) | 1.00 (ref) | 1.00 (ref) |
| 1-2 | 1.19 (1.16-1.22) | 1.24 (1.22-1.28) | 1.28 (1.25-1.31) |
| 3-5 | 1.15 (1.14-1.17) | 1.20 (1.19-1.22) | 1.25 (1.24-1.27) |
| 6-9 | 1.11 (1.09-1.14) | 1.23 (1.20-1.26) | 1.26 (1.23-1.29) |
| 10-19 | 1.22 (1.21-1.24) | 1.32 (1.30-1.33) | 1.35 (1.34-1.37) |
| ≥20 | 1.23 (1.22-1.25) | 1.39 (1.37-1.41) | 1.45 (1.43-1.48) |
| **Higher than high school** | | | |
| 0 | 1.00 (ref) | 1.00 (ref) | 1.00 (ref) |
| 1-2 | 1.19 (1.16-1.21) | 1.33 (1.30-1.37) | 1.45 (1.42-1.49) |
| 3-5 | 1.25 (1.23-1.26) | 1.40 (1.38-1.42) | 1.48 (1.46-1.50) |
| 6-9 | 1.26 (1.23-1.29) | 1.42 (1.38-1.46) | 1.46 (1.42-1.50) |
| 10-19 | 1.35 (1.33-1.36) | 1.54 (1.52-1.55) | 1.60 (1.58-1.62) |
| ≥20 | 1.44 (1.43-1.46) | 1.68 (1.65-1.70) | 1.73 (1.70-1.77) |

Adjustment for maternal age, parity, prepregnancy BMI, previous history of preterm birth, marital status, infant sex, initiation of prenatal care.
