# Supplementary material for: Maternal cigarette smoking before and during pregnancy and the risk of preterm birth: A dose–response analysis of 25 million mother–infant pairs
Source: PLoS Med. 2020 Aug 18;17(8):e1003158. doi: 10.1371/journal.pmed.1003158 (PMC7446793; doi:10.1371/journal.pmed.1003158)
Supplement: S8 Table — NVSS, National Vital Statistics System. (DOCX) [file pmed.1003158.s010.docx]

**S8 Table. The Associations of Smoking with Preterm Birth: NVSS 2014-2018**

| **Before pregnancy** | | | | | | | | | |
| --- | --- | --- | --- | --- | --- | --- | --- | --- | --- |
| **Cigarette per day** | **No. of participants, *n* (%)** | **Preterm birth (<37 weeks)** | | **Moderately preterm birth (32-36 weeks)** | | **Very preterm birth (28-31 weeks)** | | **Extremely preterm birth (<28 weeks)** | |
|  |  | **No. of Cases, *n* (%)** | **Adjusted OR (95% CI)** | **No. of Cases, *n* (%)** | **Adjusted OR (95% CI)** | **No. of Cases, *n* (%)** | **Adjusted OR (95% CI)** | **No. of Cases, *n* (%)** | **Adjusted OR (95% CI)** |
| 0 | 15,470,915 (90.7) | 1,120,389 (6.6) | 1.00 (ref) | 952,744 (5.6) | 1.00 (ref) | 94,016 (0.6) | 1.00 (ref) | 73,629 (0.4) | 1.00 (ref) |
| 1-2 | 107,140 (0.6) | 10,862 (0.1) | 1.25 (1.22-1.28) | 9,104 (0.1) | 1.25 (1.22-1.28) | 966 (0.01) | 1.25 (1.17-1.33) | 792 (0.004) | 1.26 (1.18-1.36) |
| 3-5 | 301,384 (1.8) | 30,322 (0.2) | 1.26 (1.24-1.27) | 25,217 (0.1) | 1.24 (1.23-1.26) | 2,870 (0.02) | 1.36 (1.31-1.42) | 2,235 (0.01) | 1.34 (1.28-1.40) |
| 6-9 | 111,756 (0.7) | 11,043 (0.1) | 1.24 (1.22-1.27) | 9,287 (0.1) | 1.24 (1.21-1.26) | 1,017 (0.01) | 1.33 (1.25-1.41) | 739 (0.004) | 1.25 (1.16-1.34) |
| 10-19 | 498,740 (2.9) | 50,875 (0.3) | 1.35 (1.34-1.37) | 43,047 (0.3) | 1.33 (1.32-1.35) | 4,466 (0.03) | 1.45 (1.40-1.50) | 3,362 (0.02) | 1.52 (1.47-1.58) |
| ≥20 | 573,605 (3.4) | 61,672 (0.4) | 1.42 (1.40-1.43) | 52,820 (0.3) | 1.41 (1.40-1.42) | 5,219 (0.03) | 1.47 (1.42-1.51) | 3,633 (0.02) | 1.45 (1.40-1.50) |
| **First trimester** | | | | | | | | | |
| **Cigarette per day** | **No. of participants, *n* (%)** | **Preterm birth (<37 weeks)** | | **Moderately preterm birth (32-36 weeks)** | | **Very preterm birth (28-31 weeks)** | | **Extremely preterm birth (<28 weeks)** | |
|  |  | **No. of Cases, *n* (%)** | **Adjusted OR (95% CI)** | **No. of Cases, *n* (%)** | **Adjusted OR (95% CI)** | **No. of Cases, *n* (%)** | **Adjusted OR (95% CI)** | **No. of Cases, *n* (%)** | **Adjusted OR (95% CI)** |
| 0 | 15,865,404 (93.0) | 1,150,092 (6.7) | 1.00 (ref) | 978,064 (5.7) | 1.00 (ref) | 96,404 (0.6) | 1.00 (ref) | 75,624 (0.4) | 1.00 (ref) |
| 1-2 | 100,467 (0.6) | 10,815 (0.1) | 1.32 (1.29-1.34) | 8,981 (0.1) | 1.30 (1.28-1.33) | 1,004 (0.01) | 1.38 (1.30-1.47) | 830 (0.005) | 1.41 (1.32-1.52) |
| 3-5 | 289,276 (1.7) | 31,250 (0.2) | 1.36 (1.35-1.38) | 25,936 (0.2) | 1.34 (1.32-1.36) | 2,955 (0.02) | 1.50 (1.45-1.56) | 2,359 (0.01) | 1.55 (1.49-1.62) |
| 6-9 | 95,739 (0.6) | 10,393 (0.1) | 1.38 (1.35-1.41) | 8,,769 (0.1) | 1.37 (1.34-1.40) | 945 (0.006) | 1.49 (1.39-1.59) | 679 (0.004) | 1.42 (1.32-1.54) |
| 10-19 | 438,480 (2.6) | 49,027 (0.3) | 1.49 (1.47-1.50) | 41546 (0.2) | 1.46 (1.44-1.48) | 4,384 (0.03) | 1.65 (1.60-1.70) | 3,097 (0.02) | 1.66 (1.60-1.72) |
| ≥20 | 274,174 (1.6) | 33,586 (0.2) | 1.59 (1.57-1.61) | 28,923 (0.2) | 1.59 (1.57-1.61) | 2,862 (0.02) | 1.66 (1.60-1.73) | 1,801 (0.01) | 1.48 (1.41-1.55) |
| **Second trimester** | | | | | | | | | |
| **Cigarette per day** | **No. of participants, *n* (%)** | **Preterm birth (<37 weeks)** | | **Moderately preterm birth (32-36 weeks)** | | **Very preterm birth (28-31 weeks)** | | **Extremely preterm birth (<28 weeks)** | |
|  |  | **No. of Cases, *n* (%)** | **Adjusted OR (95% CI)** | **No. of Cases, *n* (%)** | **Adjusted OR (95% CI)** | **No. of Cases, *n* (%)** | **Adjusted OR (95% CI)** | **No. of Cases, *n* (%)** | **Adjusted OR (95% CI)** |
| 0 | 16,033,999 (94.0) | 1,164,843 (6.8) | 1.00 (ref) | 989,996 (5.8) | 1.00 (ref) | 97,773 (0.6) | 1.00 (ref) | 77,074 (0.5) | 1.00 (ref) |
| 1-2 | 95,659 (0.6) | 10,992 (0.1) | 1.40 (1.37-1.43) | 9,175 (0.1) | 1.39 (1.36-1.42) | 1,029 (0.01) | 1.48 (1.39-1.58) | 788 (0.005) | 1.40 (1.30-1.50) |
| 3-5 | 291,785 (1.7) | 32,791 (0.2) | 1.43 (1.41-1.45) | 27,387 (0.2) | 1.41 (1.39-1.43) | 3,117 (0.02) | 1.60 (1.54-1.66) | 2,287 (0.01) | 1.52 (1.46-1.59) |
| 6-9 | 94,231 (0.6) | 10,409 (0.1) | 1.41 (1.38-1.44) | 8,909 (0.1) | 1.42 (1.38-1.45) | 910 (0.005) | 1.48 (1.39-1.58) | 590 (0.03) | 1.30 (1.20-1.41) |
| 10-19 | 385,539 (2.3) | 44,537 (0.3) | 1.53 (1.51-1.55) | 38,256 (0.2) | 1.52 (1.51-1.54) | 3,832 (0.02) | 1.64 (1.59-1.70) | 2,449 (0.01) | 1.49 (1.43-1.56) |
| ≥20 | 162,327 (1.0) | 21,591 (0.1) | 1.69 (1.66-1.71) | 18,496 (0.1) | 1.69 (1.66-1.71) | 1,893 (0.01) | 1.80 (1.72-1.89) | 1,202 (0.01) | 1.58 (1.49-1.67) |

Adjustment for maternal age, race/ethnicity, parity, education levels, prepregnancy BMI, previous history of preterm birth, marital status, infant sex, and initiation of prenatal care.
