# Supplementary material for: Maternal cigarette smoking before and during pregnancy and the risk of preterm birth: A dose–response analysis of 25 million mother–infant pairs
Source: PLoS Med. 2020 Aug 18;17(8):e1003158. doi: 10.1371/journal.pmed.1003158 (PMC7446793; doi:10.1371/journal.pmed.1003158)
Supplement: S9 Table — (DOCX) [file pmed.1003158.s011.docx]

**S9 Table. Sensitivity Analysis for the Associations of Timing of Maternal Smoking with Preterm Birth after Additional Adjustment for a Propensity Score**

| **Timing of Maternal Smoking** | | | **Preterm birth** |
| --- | --- | --- | --- |
| **Before pregnancy** | **Trimester 1** | **Trimester 2** | **Adjusted OR (95% CI)** |
| Yes | Yes | Yes | 1.45 (1.45-1.45) |
| Yes | Yes | No | 1.17 (1.17-1.18) |
| Yes | No | Yes | 1.09 (1.07-1.11) |
| Yes | No | No | 1.00 (1.00-1.01) |
| No | Yes | Yes | 1.36 (1.33-1.40) |
| No | Yes | No | 1.26 (1.23-1.28) |
| No | No | Yes | 1.31 (1.28-1.35) |
| No | No | No | 1.00 (ref) |

Adjustment for maternal age, race/ethnicity, parity, education levels, prepregnancy BMI, previous history of preterm birth, marital status, infant sex, initiation of prenatal care, and a propensity score that reflected the association of smoking status with other covariates.
