# Supplementary material for: Maternal cigarette smoking before and during pregnancy and the risk of preterm birth: A dose–response analysis of 25 million mother–infant pairs
Source: PLoS Med. 2020 Aug 18;17(8):e1003158. doi: 10.1371/journal.pmed.1003158 (PMC7446793; doi:10.1371/journal.pmed.1003158)
Supplement: S10 Table — (DOCX) [file pmed.1003158.s012.docx]

**S10 Table. Sensitivity Analysis for the Associations of Maternal Smoking with Preterm Birth after Additional Adjustment for a Propensity Score**

| **Before pregnancy** |  |
| --- | --- |
| **Cigarette per day** | **Adjusted OR (95%CI)** |
| 0 | 1.0 (ref) |
| 1-2 | 1.22 (1.21-1.23) |
| 3-5 | 1.23 (1.22-1.23) |
| 6-9 | 1.21 (1.20-1.22) |
| 10-19 | 1.32 (1.32-1.33) |
| ≥20 | 1.36 (1.36-1.36) |
| **First trimester** |  |
| **Cigarette per day** | **Adjusted OR (95%CI)** |
|  | 1.00 (ref) |
| 1-2 | 1.30 (1.30-1.31) |
| 3-5 | 1.31 (1.30-1.31) |
| 6-9 | 1.33 (1.32-1.34) |
| 10-19 | 1.44 (1.43-1.44) |
| ≥20 | 1.53 (1.52-1.53) |
| **Second trimester** |  |
| **Cigarette per day** | **Adjusted OR (95%CI)** |
| **0** | **1.0 (ref)** |
| 1-2 | 1.36 (1.36-1.37) |
| 3-5 | 1.36 (1.35-1.36) |
| 6-9 | 1.36 (1.35-1.36) |
| 10-19 | 1.47 (1.47-1.48) |
| ≥20 | 1.58 (1.58-1.59) |

Adjustment for maternal age, race/ethnicity, parity, education levels, prepregnancy BMI, previous history of preterm birth, marital status, infant sex, initiation of prenatal care, and a propensity score that reflected the association of smoking status with other covariates.
