# Supplementary material for: Maternal cigarette smoking before and during pregnancy and the risk of preterm birth: A dose–response analysis of 25 million mother–infant pairs
Source: PLoS Med. 2020 Aug 18;17(8):e1003158. doi: 10.1371/journal.pmed.1003158 (PMC7446793; doi:10.1371/journal.pmed.1003158)
Supplement: S11 Table — (DOCX) [file pmed.1003158.s013.docx]

**S11 Table. Sensitivity Analysis for the Association Between Smoking Cessation at Various Periods and Preterm Birth after Additional Adjustment for a Propensity Score**

| **Cigarette per day** | **Cessation before pregnancy** | **Cessation during the first trimester** | **Smoking throughout the second trimester** |
| --- | --- | --- | --- |
| Never smoking | 1.00 (ref） | 1.00 (ref） | 1.00 (ref） |
| **Before pregnancy** |  |  |  |
| 1-2 | 0.99 (0.98-1.001)* | 1.23 (1.21-1.25) | 1.46 (1.44-1.47) |
| 3-5 | 0.99 (0.99-1.00) | 1.15 (1.14-1.16) | 1.43 (1.42-1.43) |
| 6-9 | 0.97 (0.96-0.98) | 1.14 (1.12-1.16) | 1.36 (1.35-1.37) |
| 10-19 | 1.00 (0.996-1.01) | 1.15 (1.14-1.16) | 1.46 (1.45-1.46) |
| ≥20 | 1.01 (1.004-1.02) | 1.17 (1.16-1.18) | 1.46 (1.45-1.46) |
| **Trimester 1** |  |  |  |
| 1-2 | NA | 1.13 (1.11-1.14) | 1.39 (1.38-1.40) |
| 3-5 | NA | 1.12 (1.11-1.13) | 1.36 (1.35-1.36) |
| 6-9 | NA | 1.19 (1.17-1.21) | 1.35 (1.34-1.36) |
| 10-19 | NA | 1.19 (1.18-1.20) | 1.46 (1.46-1.47) |
| ≥20 | NA | 1.25 (1.24-1.27) | 1.55 (1.55-1.56) |
| **Trimester 2** |  |  |  |
| 1-2 | NA | NA | 1.38 (1.37-1.39) |
| 3-5 | NA | NA | 1.37 (1.36-1.37) |
| 6-9 | NA | NA | 1.36 (1.36-1.37) |
| 10-19 | NA | NA | 1.48 (1.47-1.48) |
| ≥20 | NA | NA | 1.59 (1.59-1.60) |

Cessation before pregnancy: smoking before pregnancy but not smoking during the first trimester and the second trimester.

Cessation during the first trimester: smoking before pregnancy and during the first trimester, but not smoking during the second trimester.

Smoking throughout the second trimester: smoking before pregnancy and throughout pregnancy

Never smoking: not smoking before and during pregnancy.

Adjustment for maternal age, race/ethnicity, parity, education levels, prepregnancy BMI, previous history of preterm birth, marital status, infant sex, initiation of prenatal care, and a propensity score that reflected the association of smoking status with other covariates.

*Adjusted OR (95% CI)
